# Associations of physical frailty with health outcomes and brain structure in 483 033 middle-aged and older adults: a population-based study from the UK Biobank

**DOI:** 10.1016/S2589-7500(23)00043-2

**Published:** 2023-04-14

**Authors:** Rongtao Jiang, Stephanie Noble, Jing Sui, Kwangsun Yoo, Matthew Rosenblatt, Corey Horien, Shile Qi, Qinghao Liang, Huili Sun, Vince D Calhoun, Dustin Scheinost

**Affiliations:** Department of Radiology and Biomedical Imaging (R Jiang PhD, S Noble PhD, D Scheinost PhD) and Child Study Center (D Scheinost), Yale School of Medicine, New Haven, CT, USA; State Key Laboratory of Cognitive Neuroscience and Learning, Beijing Normal University, Beijing, China (J Sui PhD); Department of Psychology (K Yoo PhD), Department of Biomedical Engineering (M Rosenblatt MSc, Q Liang MSc, H Sun BS, D Scheinost), Interdepartmental Neuroscience Program (C Horien PhD, D Scheinost), and Department of Statistics and Data Science (D Scheinost), Yale University, New Haven, CT, USA; Tri-institutional Center for Translational Research in Neuroimaging and Data Science, Georgia State University, Georgia Institute of Technology, and Emory University, Atlanta, GA, USA (S Qi PhD, V D Calhoun PhD)

## Abstract

**Background:**

Physical frailty is a state of increased vulnerability to stressors and is associated with serious health issues. However, how frailty affects and is affected by numerous other factors, including mental health and brain structure, remains underexplored. We aimed to investigate the mutual effects of frailty and health using large, multidimensional data.

**Methods:**

For this population-based study, we used data from the UK Biobank to examine the pattern and direction of association between physical frailty and 325 health-related measures across multiple domains, using linear mixed-effect models and adjusting for numerous confounders. Participants were included if complete data were available for all five indicators of frailty, all covariates, and at least one health measure. We further examined the association between frailty and brain structure and the role of this association in mediating the relationship between frailty and health outcomes.

**Findings:**

483 033 participants aged 38–73 years were included in the study at baseline (between Dec 19, 2006, and Oct 1, 2010); at a median follow-up of 9 years (IQR 8–10), behavioural data were available for 46 501 participants and neuroimaging data for 40 210 participants. The severity of physical frailty was significantly associated with decreased cognitive performance (Cohen’s *d*=0·025–0·162), increased early-life risks (*d*=0·026–0·111), unhealthy lifestyle (*d*=0·013–0·394), poor physical fitness (*d*=0·007–0·668), increased symptoms of poor mental health (*d*=0·032–0·607), severe environmental pollution (*d*=0·013–0·064), and adverse biochemical markers (*d*=0·025–0·198). Some associations were bidirectional, with the strongest effects on mental health measures. The severity of frailty correlated with increased total white matter hyperintensity and lower grey matter volume, particularly in subcortical regions (*d*=0·027–0·082), which significantly mediated the association between frailty and health-related outcomes, although the mediated effects were small.

**Interpretation:**

Physical frailty is associated with diverse unfavourable health-related outcomes, which can be mediated by differences in brain structure. Our findings offer a framework for guiding preventative strategies targeting both frailty and psychiatric disorders.

## Introduction

Physical frailty results in increased vulnerability to stressors due to deterioration in functional reserve across various physiological systems.^1^ Frailty is common in older adults and places individuals at a greater risk of adverse outcomes, including disability, diminished quality of life, and premature mortality.^2–4^ Many clinical guidelines now advocate for the routine monitoring of frailty.

Despite the clinical and public health implications of frailty,^5^ existing work relating frailty to health-related outcomes is fragmented and limited by the scarcity of data resources with the appropriate scale and breadth of health measures. Previous literature on frailty has primarily focused on characterising its correlations with aspects of physiological capabilities and morbidities, leaving its association with numerous other health-related outcomes largely unexplored. Further, emerging evidence suggests that changes in brain reserve can contribute to the occurrence and progression of frailty during ageing.^6–8^ For example, established hallmarks of brain ageing—grey matter volume and total white matter hyperintensity—have been linked to broad physical and mental health measures. However, to date, only a few small studies have examined the neurobiological underpinnings of physical frailty, with conflicting findings.^9,10^ Discrepancies in these findings can be attributed to heterogeneity in sample characteristics, differences in adjustment for covariates, and small sample sizes.^11,12^ Moreover, given that most evidence is cross-sectional, the directionality of the association is unclear. Does physical frailty predict future health outcomes, or are these health measures risk factors for future frailty? Similarly, the mediative role of brain structure in linking frailty and multiple health outcomes remains unknown.

A large, multidimensional, longitudinal dataset is needed to answer these questions; however, such data have not been available so far. We characterised the association of physical frailty with a comprehensive set of health outcomes and neuroimaging data from approximately 500 000 participants in the UK Biobank.^13^

## Methods

### Study design and participants

This study used data from the UK Biobank, one of the largest prospective, population-based cohorts recruited from across the UK.^13^ Between 2006 and 2010, demographic and health-related information was collected from more than 500 000 participants at 22 dedicated assessment centres (baseline visits). Since 2014, a subsample of participants has been invited back for neuroimaging and a repeat assessment of phenotypic information, including frailty (imaging visit). Only participants with complete data available for the five indicators of frailty (slow walking speed, weight loss, weakness, exhaustion, and physical inactivity), all covariates (age, sex, BMI, education level, waist-to-hip ratio, ethnicity, and material deprivation), and at least one health measure were included (appendix 1 p 1).

Unlike previous analyses that focused on specific health or behavioural domains, we used health outcomes and measures across multiple domains (eg, cognition, mental and physical health, environment, and lifestyle). Such an approach enables the discovery of previously unanticipated factors relating to frailty and the comparison of the relative importance of multiple domains simultaneously. Using the most extensive sample available affords statistical power to capture frailty-related exposures or outcomes with minor to moderate effects.^11^ Longitudinal analyses help to elucidate the temporal relationship between frailty and health outcomes to inform health-care policies and facilitate the development of targeted interventions.

### Assessment of physical frailty

The Fried frailty phenotype^14^ and Rockwood frailty index^15^ are widely used in frailty research. We used the Fried frailty phenotype in this study because its indicators (eg, physical activity, grip strength, and weight) are modifiable and easy to collect, which could have important implications for designing and planning interventions. Traits constituting the Rockwood frailty index are mainly health deficits and largely overlap with the health measures we planned to examine; using this index could therefore prevent us from establishing the relevance of frailty to numerous health outcomes. The Fried frailty phenotype has been validated in many studies associated with mortality,^3^ multimorbidity,^3^ dementia,^16^ and severe COVID-19^17^ that make use of UK Biobank data.

The Fried frailty phenotype assesses the following five indicators: weight loss, exhaustion, weakness, physical inactivity, and slow walking speed. The number of criteria met indicated the severity of frailty, resulting in a score from 0 to 5. Following other validated versions of frailty assessment,^3,17^ we adapted the definitions of some items to accommodate the UK Biobank data. Weight loss was derived from the self-reported weight change in the past year, and dichotomised into yes (lost weight) or no (same weight or gained weight). Exhaustion was defined as answering “More than half the days or nearly every day” to the question “Over the past 2 weeks, how often have you felt tired or had little energy?” Walking speed was categorised as slow or normal (steady or brisk) on the basis of self-reported walking pace. Physical activity was assessed on the basis of responses to the question about activity types and frequency in the past 4 weeks and classified as physically inactive (no or light activity with a frequency of once per week or less) or physically active (medium or healthy activity or light activity more than once per week). Weakness was defined as low mean grip strength in both the left and right hands, using sex and BMI-adjusted cutoffs. A detailed description can be found in appendix 1 (p 2) and elsewhere.^3,16^

### Health-related measures

We identified 325 health-related measures and outcomes (an extension of measures used in a previous study^18^) for which data were available for at least 10 000 participants. A complete list of these measures can be found in appendix 2. The measures can be grouped into seven broad categories: cognitive functioning (n=21; eg, fluid intelligence, executive function, and processing speed); lifestyle factors (n=91; eg, smoking status, alcohol, sleep, diet, and electronic device use); early-life risks (n=10; eg, maternal smoking, birth weight, and history of breastfeeding); physical health (n=62; eg, blood pressure, fat mass, pain, and body composition); mental health (n=82; eg, depression, trauma, self-harm, mania, anxiety, wellbeing, life satisfaction, and social support—a large proportion of these measures were assessed through a detailed and comprehensive mental health questionnaire online, which was developed by the UK Biobank); biochemical markers (n=30; eg, C-reactive protein, IGF-1, and albumin); and environmental factors (n=29; eg, air pollution, noise pollution, proximity to greenspace, and distance to road). Most of these measures were self-reported and responses were collected by questionnaires on touchscreen devices at the assessment centre or through online questionnaires.

### MRI data acquisition and processing

All brain MRI data were acquired on a 3T Skyra scanner (Siemens), preprocessed and quality controlled, and made available to approved researchers as image-derived phenotypes. The grey matter volumes of 139 cortical and subcortical regions (appendix 1 p 3) and total white matter hyperintensity were extracted. An extensive overview of the data acquisition, protocols, and preprocessing is available online.

Extreme outlying data points (outside 5 SD), which could result from processing errors or severe brain irregularities, were excluded on a case-by-case basis.^19^ Neuroimaging measures were adjusted for head size by multiplying raw volumes by the head scaling factor. Total white matter hyperintensity was further log-transformed to normalise and stabilise the variance.^20^

### Statistical analysis

We used linear mixed-effect models to investigate the associations between physical frailty and health outcomes.^21,22^ Separate models were fitted for the 325 health measures within the same analytical framework. Within each model, our primary objective was to characterise how the severity of frailty relates to the health measures while simultaneously adjusting for numerous confounders. Therefore, the health-related measure was the dependent variable and the severity of frailty was an independent fixed effect. The model also included age, sex, BMI, education level, waist-to-hip ratio, ethnicity (White or non-White), and material deprivation as additional covariates of no interest. The data acquisition site was included as a random effect to account for non-independence between distinct imaging centres and other potentially associated variables, as done previously.^21^ We extracted the standardised coefficient (β) and associated two-tailed p value from each model. We fitted a generalised linear mixed-effect model with binomial error structure and logit link function and obtained the log-transformed odds ratio (OR) for binary variables. To facilitate comparisons of effect sizes across all measures, we converted β for continuous measures and log-transformed OR for binary measures to Cohen’s *d* according to a previous study^23^ using the effectsize package in R (version 4.1.2). We compensated for multiple comparison for all 325 p values using Bonferroni correction.

To ensure robust results we conducted sensitivity analyses, in which the models were stratified by sex and age category (midlife 45–60 years, old >60 years; to ensure a similar number of samples between subgroups, we excluded participants aged 45 years and younger). A sensitivity analysis was done by additionally incorporating average total household income, smoking status, and frequency of alcohol intake as covariates.

The same analytical framework was used to assess the associations of regional grey matter volumes and total white matter hyperintensity with physical frailty and the top ten measures showing significant correlations with physical frailty. In addition to the confounders listed, we included the total intracranial volume as a covariate. To establish whether physical frailty and these health-related measures have brain correlates in common, we calculated the Pearson’s correlation coefficient of the association maps between frailty and each health measure. Multiple comparisons were corrected using the Benjamini-Hochberg false discovery rate (FDR) method.

Of all 325 health measures, 152 had longitudinal data acquired at the imaging visit over a median 9-year follow-up, covering cognition, lifestyle, physical measures, and mental health. Health measures available for fewer than 5000 participants were excluded from the longitudinal analyses. We followed a similar linear or generalised mixed-effect model approach to characterise how baseline frailty related to the 152 health measures at follow-up. Specifically, within each model, the health measure collected 9 years later was modelled as the response variable, baseline frailty as the independent variable, and data sites were modelled as random effects. The same covariates used in association analyses, their changes between the baseline and follow-up assessments, and health measures at baseline were modelled as fixed effects of no interest. Similar analyses were done to evaluate the reverse associations between baseline health measures and the progression of frailty at follow-up.

We used the mediation package in R (version 4.1.2) to establish how much of the covariance between frailty and health outcomes can be explained by brain structure.^22^ For each health measure, we established a standard three-variable path model, in which frailty was used as the independent variable, health outcome as the dependent variable, and log-transformed total white matter hyperintensity or mean grey matter volume of brain regions that were significantly correlated with both frailty and the health measure as the mediator. Confounding variables were regressed out in the mediation analysis. Given the bidirectional relationship between frailty and these health outcomes, we ran additional mediation analyses by switching the role of independent and dependent variables. 10 000 bootstrap iterations were used to assess the significance of mediation effects.

### Role of the funding source

The funder had no role in the study design, data collection, data analysis, data interpretation, or writing of the report.

## Results

**Figure 1:**
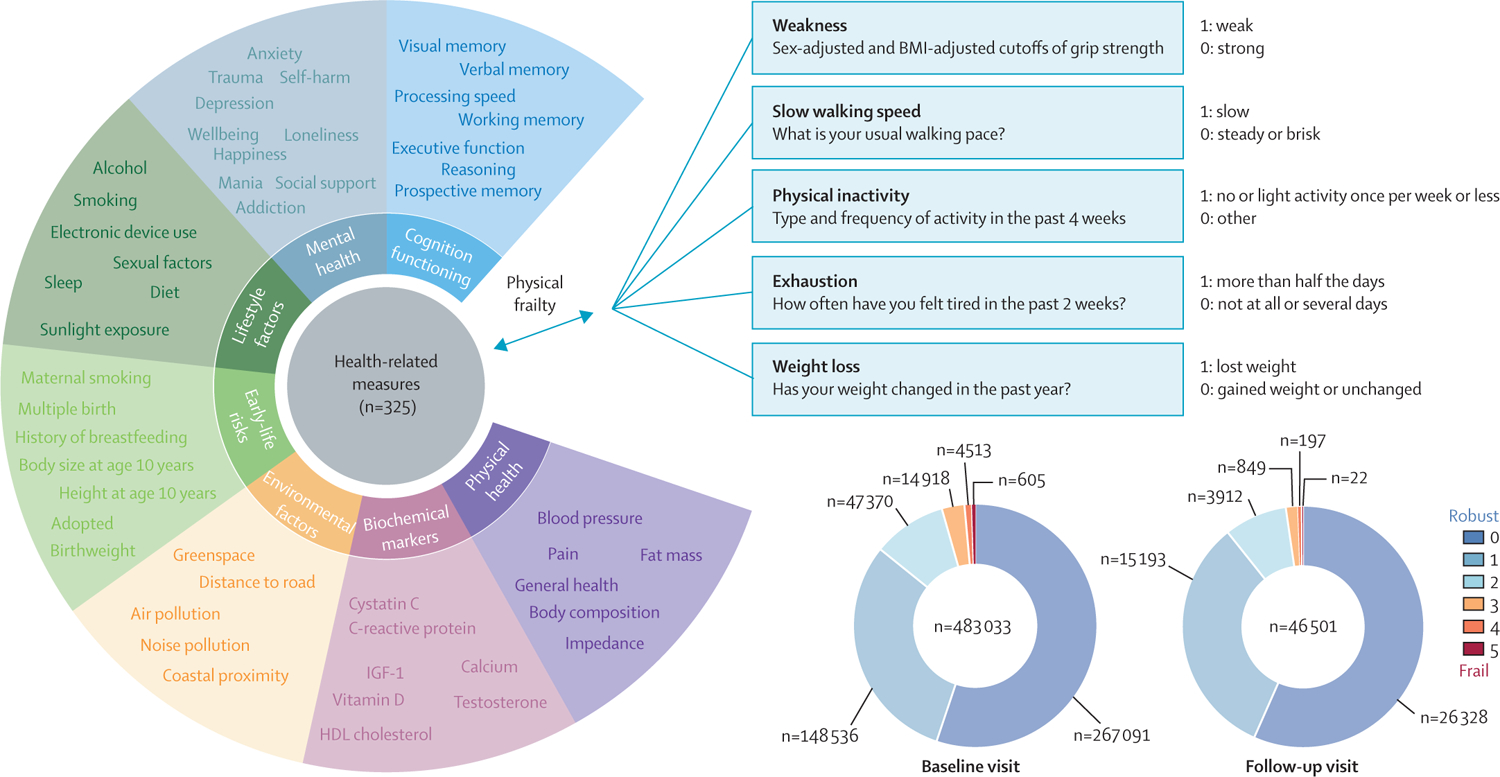
Summary of health outcomes and four analyses performed in the present study Top, the 325 health-related measures assessed are grouped into seven broad categories. Bottom, the number of participants with the indicated number of frailty indicators (physical frailty score) at baseline and 9-year follow-up.

The study sample included up to 483 033 participants (12 532–483 033 per specific health measure) who, at the baseline visit, had complete data available for all five frailty indicators, all covariates, and at least one health-related measure (figure 1). The mean age was 56 years (SD 8; range 38–73). 262 784 (54·40%) participants were female and 457 357 (94·68%) were White. After a 9-year follow-up, 46 501 (9·62%) participants had behavioural data for longitudinal analyses (mean age 64·12 years [SD 7·73, range 44–82]; 5228–45 515 per specific health measure) and 40 210 (8·32%) participants had grey matter volume data for neuroimaging analyses (40 124–40 206 per specific brain region). We summarised the demographic characteristics and missingness of participants (appendix 1 pp 4–7) and the number of participants with varying frailty indicators and their fluctuation over time (appendix 1 pp 8–9).

**Figure 2:**
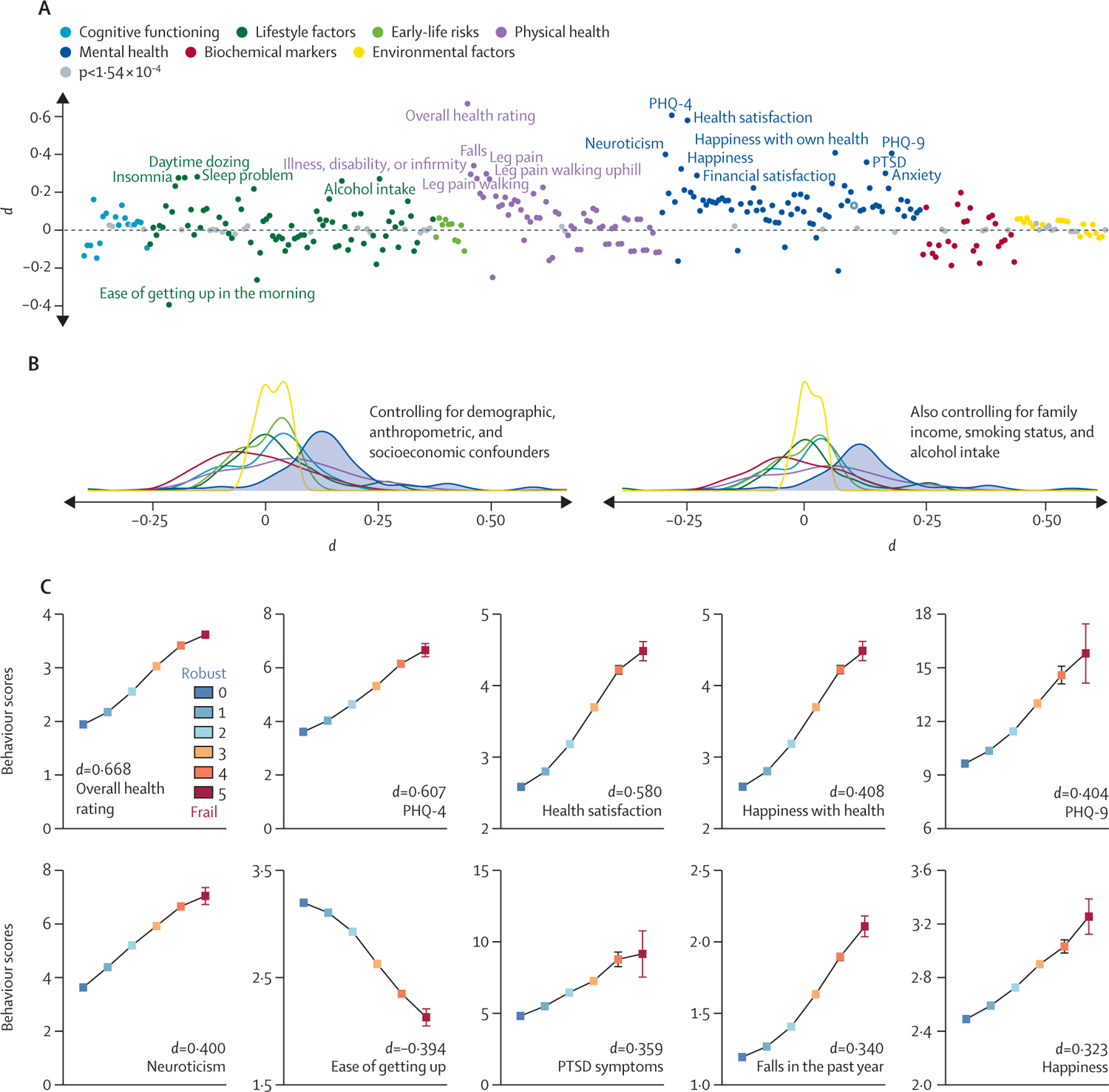
Associations between physical frailty and health-related measures (A) Of 325 health measures, 283 showed significant associations (p<1·54 × 10^−4^; Bonferroni-corrected) with physical frailty while covarying for a set of potential confounders. Phenotypes that did not show significant associations are shown in grey. The top 20 phenotypic measures showing the numerically most significant associations with frailty are highlighted. (B) Distribution of effect sizes with and without additional control for family income, smoking status, and alcohol intake. (C) The top ten health measures exhibiting the numerically most significant associations with frailty. For ease of getting up, higher values represent better outcomes; for all other measures, higher values represent worse outcomes. Data are mean (95% CI). PHQ=patient health questionnaire. PTSD=post-traumatic stress disorder.

283 (87%) of 325 health-related measures had Bonferroni-significant associations (p<1·54 × 10^−4^; effect sizes ranged from *d*=0·007 to *d*=0·668) with physical frailty while controlling for numerous confounders (figure 2A). All associations were in the expected direction, with the severity of frailty being related to decreased cognitive performance, increased early-life risks, unhealthy lifestyle, poor physical fitness, increased symptoms of poor mental health, severe environmental pollution, and adverse biochemical markers. In particular, larger effect sizes were seen for the associations with mental health measures than for other categories (figure 2B); 78 of 82 mental health traits we examined reached significance.

The top ten health measures exhibiting the numerically most significant associations with frailty were overall health rating (*d*=0·668, 95% CI 0·662 to 0·673, n=481 088), Patient Health Questionnaire 4 (PHQ-4, which measures severity of depression; 0·607, 0·599 to 0·613, n=442 740), health satisfaction (0·580, 0·570 to 0·590, n=165 814), PHQ-9 (severity of depression measured online; 0·404, 0·393 to 0·415, n=151 525), happiness with own health (0·408, 0·398 to 0·419, n=153 991), neuroticism (0·400, 0·393 to 0·406, n=389 668), ease of getting up in the morning (−0·394, −0·400 to −0·389, n=482 430), post-traumatic stress disorder symptoms (0·359, 0·343 to 0·374, n=69 086), falls (0·340, 0·335 to 0·347, n=482 072), and general happiness (0·323, 0·314 to 0·335, n=165 736; figure 2C). Notably, seven of these ten were mental health measures. The exhaustion component was excluded from PHQ-4 and PHQ-9 (appendix 1 p 10).

The subgroup analyses yielded nearly identical findings to the main results, with the pattern of associations highly correlated between the sexes and between middle-aged and older adults (p<10^−10^, appendix 1 pp 11–12) across all categories. An exception is the mental health measures, for which middle-aged people showed more significant associations (in terms of effect size and proportion of significant associations) than their older counterparts. Sensitivity analysis revealed nearly unchanged results when additionally controlling for confounders (appendix 1 p 13) or treating frailty phenotype as a categorical variable (appendix 1 p 14). Moreover, the associations between physical frailty and health-related outcomes were not driven by any single indicator. Each indicator showed independent associations with most health measures, with exhaustion and slow walking speed being the most significant indicators and weight loss being the least significant indicator (appendix 1 pp 15–17).

**Figure 3:**
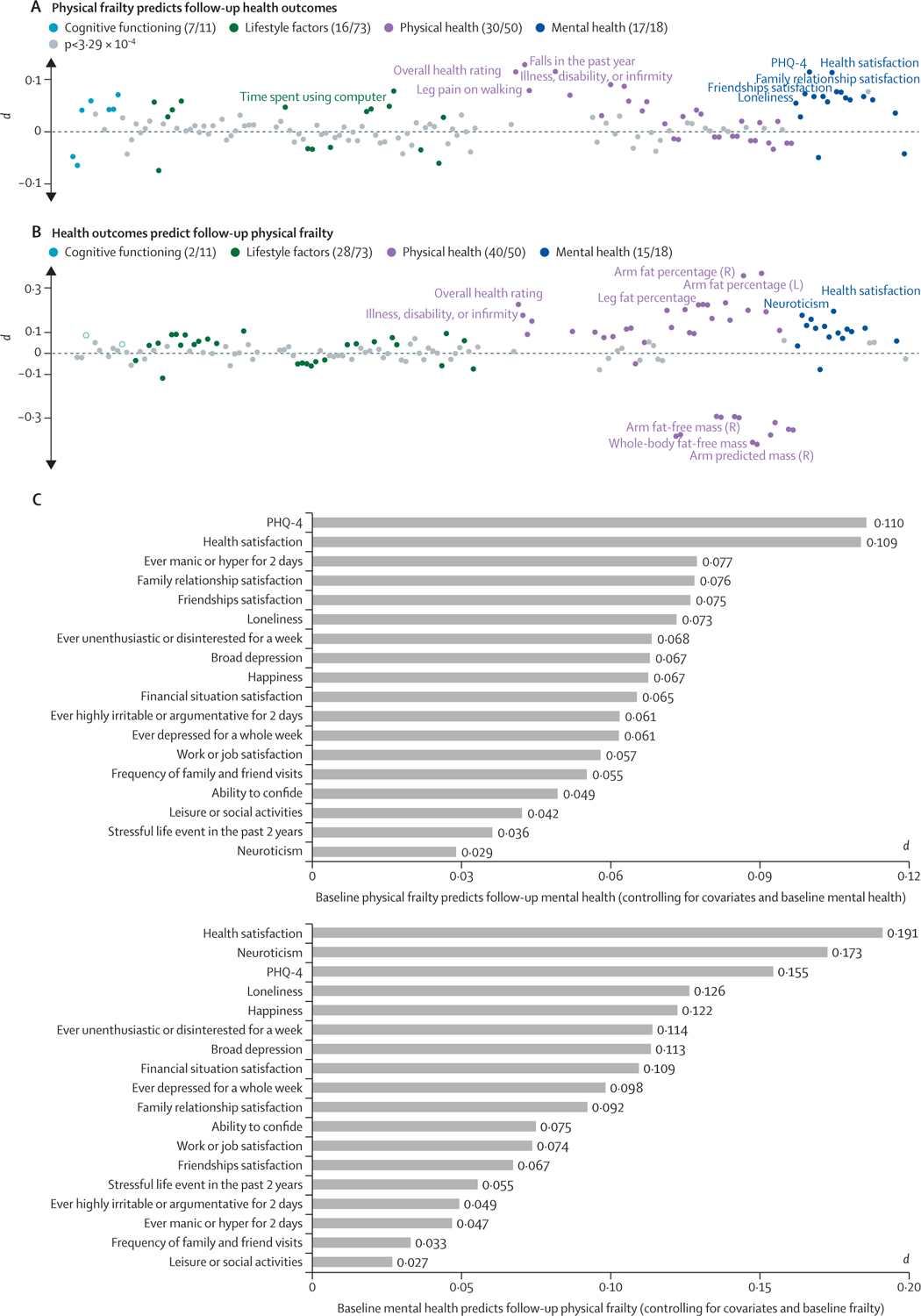
The longitudinal association between physical frailty and health-related measures (A) Physical frailty at baseline was significantly associated with 70 of 152 health-related outcomes examined at follow-up after controlling for the baseline level of these outcomes and numerous confounders alongside their change over time (p<3·29 × 10^−4^; Bonferroni-corrected). The pie charts show the number of significant and insignificant associations. Health measures not showing significant associations are shown in grey. The top ten health measures showing numerically significant associations with frailty are highlighted. (B) The reverse associations (baseline health measures predicting future frailty) reached statistical significance for 85 measures. (C) Significant bidirectional associations were observed for mental health measures, for which baseline frailty predicted 17 of 18 mental health traits at a 9-year follow-up, and the reverse was also significant in 15 of 18 measures. L=left. R=right. PHQ=patient health questionnaire.

20 355 (44·6%) of 45 661 participants had at least one change in the severity of frailty score over time, including both worsening and improvement (appendix 1 p 9). Overall, physical frailty at baseline significantly predicted about half (70 of 152) of the phenotypic outcomes at 9-year follow-up after controlling for the baseline level, confounders, and the change in these variables (p<3·29 × 10^−4^, the Bonferroni-corrected level of association). All associations were in the expected direction: severe frailty related to decreased cognitive performance, poorer physical fitness, and increased symptoms of poor mental health. Among all four categories—cognition, lifestyle, physical measures, and mental health—the largest proportion of significant associations was found for mental health measures (17 of 18 were significant; figure 3A).

The reverse associations (ie, health outcomes predicting frailty) reached significance for 85 of 152 measures (p<3·29 × 10^−4^; figure 3B). The highest significance rate was again observed for mental health measures (15 of 18 were significant), suggesting that having more severe mental health symptoms is linked to more severe frailty at the 9-year follow-up. These results suggest that a bidirectional relationship might exist between frailty and adverse health outcomes, especially those relating to mental health.

We found widespread associations between physical frailty and regional grey matter volumes across cortical, subcortical, and cerebellar regions (appendix 1 p 18). The strongest associations were observed in the accumbens, thalamus, hippocampus, brain stem, anterior temporal fusiform cortex, parahippocampus, cerebellum VIIIa, temporal pole, and pallidum. After controlling for confounders, the total white matter hyperintensity had a significantly positive correlation with the severity of frailty (*d*=0·084, P_FDR_=7·71 × 10^−21^). Specifically, the severity of frailty was significantly and negatively related to grey matter volume in 75 brain regions (|*d*|=0·027 – 0·082, P_FDR_<0·01, appendix 1 pp 19–20), independent of confounding factors.

Each of the top ten frailty-related health measures also exhibited widespread associations with regional grey matter volumes across the whole brain, although the number of brain regions for which these associations reached significance varied (appendix 1 p 21). In addition, all ten measures had a significant correlation with the total white matter hyperintensity (|*d*|=0·020 – 0·116, P_FDR_<0·05, appendix 1 p 22).

Further, the association map of frailty was significantly similar to eight of the top ten frailty-correlated health outcomes (P_FDR_<0·05, appendix 1 p 18). The similarity ranged from |*r*|=0·19 to |*r*|=0·61. These results suggest that frailty and these health outcomes could have shared neurobiological correlates.

The mediation effect of brain structure on the association between frailty and each of the top ten frailty-correlated health measures, after adjusting for confounders, is shown in appendix 1 (p 18). Specifically, the mean grey matter volume significantly and partially mediated the effect of frailty on all ten health-related measures or the effect of these measures on frailty, with the proportion of mediated variance ranging from 0·23% to 1·59% (P_FDR_<0·05, appendix 1 p 23).

The mediation analysis also revealed a partial but significant indirect effect of log-transformed total white matter hyperintensity on the association between frailty and health outcomes in both directions, with the exception of the outcome ease of getting up (P_FDR_<0·05, appendix 1 p 24). The mediated effect varied between 0·27% and 1·40%. These results suggest that the relationship between physical frailty and multiple health-related outcomes could be explained by differences in brain structure (grey matter volume and total white matter hyperintensity).

## Discussion

Using data from nearly 500 000 participants, we showed that physical frailty was associated with a diverse set of unfavourable outcomes, with the most substantial effects observed for mental health outcomes. We also found that the severity of frailty was correlated with elevated total white matter hyperintensity and lower grey matter volume, particularly in subcortical brain regions. Furthermore, we established that brain structures significantly and partially mediated the association between frailty and these health-related measures.

Positive associations between the severity of frailty and adverse health outcomes concur with previous studies of physical frailty in older adults. The breadth of data in this study provides sufficient statistical power to accurately estimate the effect size, resulting in the identification of risk factors or consequences of frailty that—to our knowledge—have not yet been discussed. Across all analyses, mental health measures were as significant or even more significant than physical measures, suggesting that people with physical frailty could be at increased risk of mental health symptoms and vice versa. Because both frailty and mental health measures were self-reported, these results suggest that self-perceived physical frailty is highly correlated with a person’s subjective feelings of depression, anxiety, happiness, life satisfaction, and loneliness. This finding is consistent with a few epidemiological studies and meta-analyses in smaller samples.^24,25^ A 2021 study of 9171 participants from the English Longitudinal Study of Ageing reported that loneliness and social isolation were independently associated with increasing frailty, indicating a synergistic interaction between frailty and social functioning.^26^ Longitudinal analyses provided further evidence that some of these associations were bidirectional, especially for mental health, reflecting an increase in mental health symptoms after baseline physical frailty is reached and the subsequent progression of frailty following poor mental health over a 9-year interval. The bidirectional association offers a framework for guiding preventative strategies targeting both physical frailty and mental health symptoms, such that interventions that endorse either physical capabilities or mental health could have the capacity to benefit the other.

The subgroup analyses suggest a higher association magnitude in people younger than 60 years than in their older counterparts. Despite the focus of frailty research on older participants,^5^ this finding suggests that physical frailty could be important in midlife and an indicator of future poor mental health in old age. Our finding argues for increased attention to early surveillance and intervention, which could benefit middle-aged adults in the prodromal stage of frailty. Nevertheless, these findings should be interpreted with caution given that scant information is available on the degree to which frailty represents the same construct between different age groups. For example, we have only self-reported weight loss status rather than the initially defined measure of unintentional loss of at least 5% of bodyweight. As such, self-reported weight loss might have different health implications across age groups. For example, younger adults might be more likely to lose weight deliberately, representing a healthy indicator.^3^ Nevertheless, our sensitivity analysis indicated that the associations between weight loss and health-related measures were highly similar between age groups (appendix 1 p 25).

Although only a few studies have examined how frailty relates to grey matter volumes, our findings agree with previous work documenting associations between brain structure and frailty.^6–10^ Our neuroimaging analysis revealed that more severe physical frailty is accompanied by elevated total white matter hyperintensity, which aligns with earlier studies showing that cerebral vascular damage and brain atrophy were prominent indicators of frailty and reflected the general status of physical health.^7,27^ Moreover, nearly 50% of all brain regions showed significant negative associations between grey matter volumes and the severity of frailty, with the most significant areas including the thalamus, accumbens, cerebellum, temporal pole, and hippocampus. The crucial role of these subcortical regions in frailty is consistent with their involvement in the regulation of cognitive and sensorimotor processes.^28^ Grey matter volume and total white matter hyperintensity significantly mediated the associations between physical frailty and health-related outcomes, raising the possibility of a neurobiological basis. Potential mechanisms include chronic inflammation, hormonal dysfunction, nutritional deficiency, and oxidative stress. Specifically, increased concentrations of proinflammatory cytokines could influence frailty by promoting protein degradation and affecting metabolic pathways in the brain.^29^ The brain can also interact with the endocrine system via the hypothalamic–pituitary axis, where abnormal expression of some essential hormones has been reported.^1,29^ Future work should test these hypotheses.

We note that, despite reaching significance, some of the associations and mediation effects were small. These results can be ascribed to the adequate adjustment for numerous demographic, anthropometric, and socioeconomic confounders that can co-occur with frailty, health outcomes, and brain morphometry. As such, the effect sizes should be interpreted as the variance beyond what can be explained by these covariates. Grey matter volumes only accounted for a small part of the neurobiological basis of frailty, and other aspects of the brain, such as the white matter microstructure, could also contribute (appendix 1 p 26). The small effect sizes further corroborate the power of using the largest sample possible to detect subtle effects that might be undetectable using smaller samples.^11^ Nevertheless, the small effect sizes can also be essential, as they convey exactly how much a specific health measure relates to frailty and can help to establish guidelines when considering a large population.^30^

Some potential limitations should be acknowledged. First, our association and mediation analyses relied on a cross-sectional study design, which can be influenced by cohort effects and does not allow for causal inferences about the relationship between physical frailty, health outcomes, and brain structure without further validation using randomised controlled trials. Nevertheless, our analysis offers a first step for future studies to examine the neurobiological mechanisms underlying frailty. Second, definitions of some frailty criteria were adapted for the data available in the UK Biobank. Four of the five frailty indicators and some health measures were self-reported,^3,16^ which might be less accurate than using objective measures. The possible reporting and recall bias could underestimate the associations. However, this concern is lessened because a trained nurse supported participants in providing self-reported data. The participants could respond that they preferred not to answer for each question. Evidence has shown that self-reported frailty was comparable with objectively measured alternatives in predicting incident disability, falls, and mortality.^31^ Additionally, objective measurements of frailty can be challenging in routine primary care practice, because they are complex and more time-consuming to collect. However, replicating the current findings deserves further examination in studies that use more precise and objective measures to assess physical frailty. Third, each indicator had equal weight in modelling the frailty phenotype. However, they might represent distinct biological constructs. A weighted combination of individual indicators of frailty might better capture health-related outcomes. Fourth, some of the data on mental health measures were acquired online several years after the baseline frailty assessment and such symptoms are likely to worsen over time; our results could therefore underestimate the magnitude of the associations of these traits with frailty. Fifth, the UK Biobank is not representative of the UK population, and the demographics of participants from whom imaging samples were obtained were different from those of the overall cohort (appendix 1 p 27), resulting in possible selection bias. Therefore, summary statistics concerning the prevalence of frailty might not generalise to the wider UK population. Nevertheless, the findings are valid for providing scientific inferences of associations between frailty and health-related outcomes. The excluded participants could introduce bias, as the missingness might not occur at random (appendix 1 p 7). Sixth, whether cognitive impairment should be included in the frailty phenotype is a highly contentious subject of debate. Investigating how frailty interacts with cognitive reserve and influences health could be a subject of interest for future research.^6^ Finally, owing to high complexity, some health-related measures in the UK Biobank that are obtained from electronic medical records—such as hospital admission, mortality registers, and primary care—were not examined here, although they merit future investigation.

Overall, this study used a population-based sample of adults in midlife and older to provide a well powered characterisation of associations between the severity of physical frailty and health-related outcomes (especially mental health outcomes) and provides insights into the neurobiological basis linking frailty to these health outcomes. The current study emphasises the importance of frailty—especially in middle-aged adults, whose mental health might be more affected by frailty than that of their older counterparts.

## Supplementary Material

1

2

## Data Availability

All data used in this study are publicly accessible from the UK Biobank via their standard data access procedure at https://www.ukbiobank.ac.uk/. Researchers can apply for access to the UK Biobank data via the Access Management System (https://www.ukbiobank.ac.uk/enable-your-research/apply-for-access).

## Research in context

### Evidence before this study

We searched PubMed for articles published in English between Jan 1, 2000, and July 1, 2022, using the search terms “frailty”, “health”, “brain structure”, “cognitive functioning”, and “mental health”; terms were searched in isolation and in combination. Physical frailty is associated with many adverse health issues, including disability, impaired quality of life, and premature mortality. However, how physical frailty affects and is affected by numerous other measures—including mental health—remains underexplored, and the findings of the few studies that examined the neurobiological underpinnings of physical frailty are largely inconsistent, possibly due to small sample sizes.

### Added value of this study

We show significant associations of physical frailty with increased total white matter hyperintensity and lower grey matter volume, which significantly mediated the relationship between frailty and health outcomes. Using data from 483 033 participants from the UK Biobank, we found that more severe physical frailty was significantly associated with decreased cognitive performance, increased early-life risks, unhealthier lifestyle, poor physical fitness, increased symptoms of poor mental health, severe environmental pollution, and adverse biochemical markers. People younger than 60 years were more strongly affected by frailty than their older counterparts, particularly in terms of mental health measures, and some associations were bidirectional. Previous literature relating frailty to health-related outcomes is fragmented and focuses on older adults. Our findings show that frailty is associated with diverse unfavourable health-related outcomes, and argue for increased attention to early surveillance and intervention, which could benefit middle-aged adults in the prodromal stage of frailty.

### Implications of all the available evidence

Our findings suggest that physical frailty could be adopted as an adjunct to traditional organ-specific diagnoses in the health-care system, and routine frailty assessment should include middle-aged adults. The neurobiological understanding of mechanisms linking physical frailty to health outcomes, including mental health, has important implications in facilitating the development of targeted interventions to prevent and counteract frailty and its adverse effects.
