# Supplementary material for: Associations of physical frailty with health outcomes and brain structure in 483 033 middle-aged and older adults: a population-based study from the UK Biobank
Source: Lancet Digit Health. Author manuscript; Available in PMC 2023 Jun 11. (PMC10257912; doi:10.1016/S2589-7500(23)00043-2)
Supplement: 1 [file NIHMS1904457-supplement-1.pdf]

THE LANCET
Digital Health

Supplementary appendix 1

This appendix formed part of the original submission and has been peer reviewed. We post it as supplied by the authors.

Supplement to: Jiang R, Noble S, Sui J, et al. Associations of physical frailty with health outcomes and brain structure in 483 033 middle-aged and older adults: a population-based study from the UK Biobank. *Lancet Digit Health* 2023; published online April 13. https://doi.org/10.1016/ S2589-7500(23)00043-2.

Associations of physical frailty with health outcomes and brain structure in 483,033 middle-aged and older adults: a population-based study from the UK Biobank

Appendix

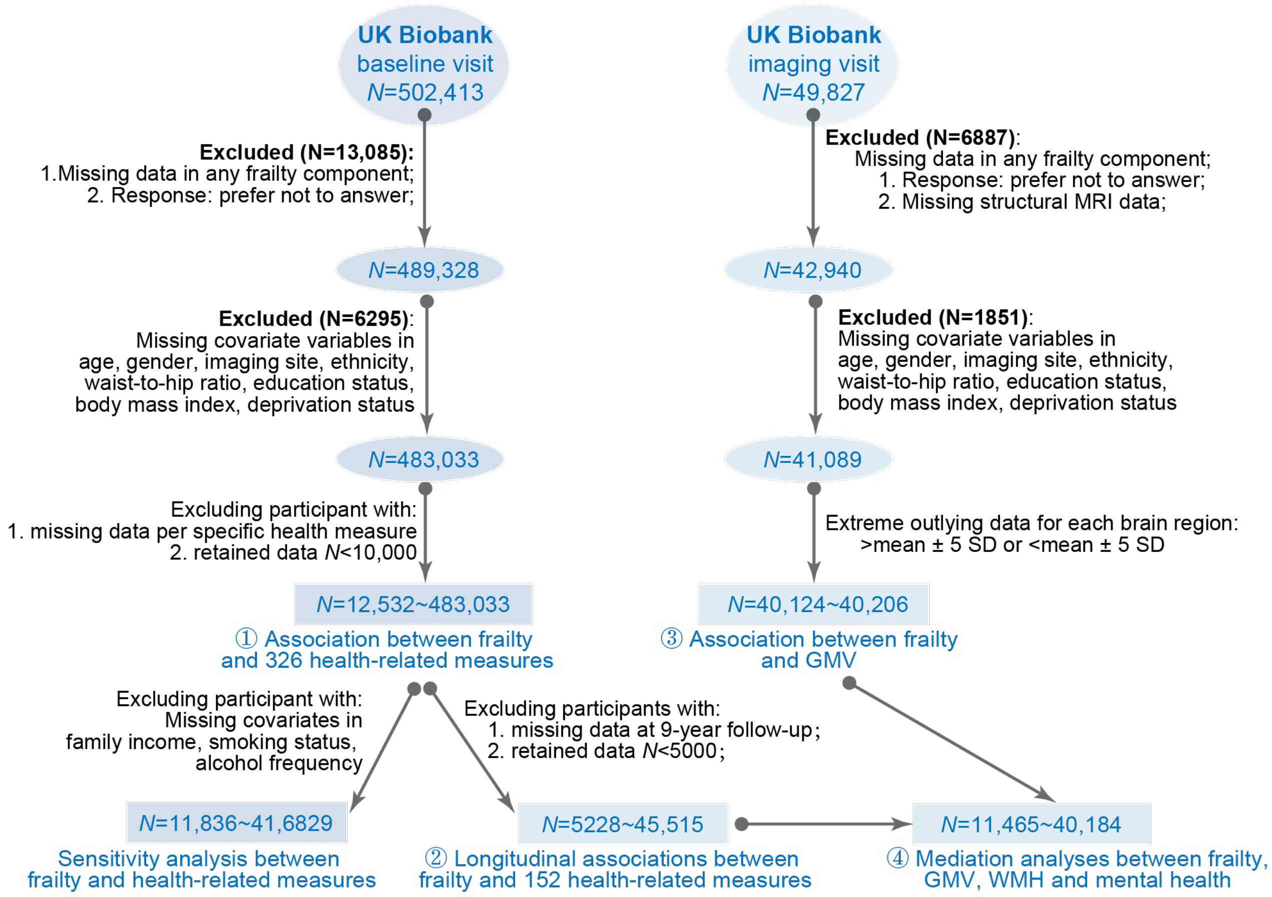

Figure S1. Flowchart illustrating criteria for selection of samples for each of the four primary analyses performed in the present study. Overall, we included up to 483,033 participants for the cross-sectional association analysis (N ranged from 12,532 to 483,033 per specific health-related measure), 46,501 participants for longitudinal association analyses (N ranged from 5,228 to 45,515 per specific health-related measure), and 40,210 participants for the imaging association analyses (N ranged from 40,124 to 40,206 per specific regional GMV).

Table S1. Definition of frailty

Frailty indicators	UK Biobank question	Field ID
Weight loss	Question: Compared with one year ago, has your weight changed?<br>· Yes - lost weight=1;<br>· No - weigh about the same=0;<br>· Yes - gained weight=0;<br>· Do not know=0;<br>· Prefer not to answer=excluded	2306
Exhaustion	Question: Over the past two weeks, how often have you felt tired or had little energy?<br>· Not at all=0;<br>· Several days=0;<br>· More than half the days=1;<br>· Nearly every day=1;<br>· Do not know=0<br>· Prefer not to answer=excluded	2080
Walking speed	Question: How would you describe your usual walking pace?<br>· Slow pace=1;<br>· Steady average pace=0;<br>· Brisk pace=0;<br>· None of the above/Prefer not to answer=excluded	924
Weakness	Grip strength was assessed isometrically using a calibrated J00105 hydraulic hand dynamometer (Lafayette Instrument Company, IN, USA), separately in the left and right arms. The average of the right and left measurement was used here, or the available one when either measurement is missing[1].<br>Cut-offs used to define low grip strength:<br>· Males:<br>BMI≤24 & grip strength≤29;<br>24.1≤BMI≤28 & grip strength≤30;<br>BMI>28 & grip strength≤32<br>· Females:<br>BMI≤23 & grip strength≤17;<br>23.1≤BMI≤26 & grip strength≤17.3;<br>26.1≤BMI≤29 & grip strength≤18;<br>BMI>29 & grip strength≤21	46; 47
Physical activity	Question: In the last 4 weeks did you spend any time doing the following?<br>· Walking for pleasure =0;<br>· Strenuous sports=0;<br>· Light DIY (*eg*: pruning, watering the lawn):<br>Frequency of once per week or less=1;<br>Frequency of more than once per week=0;<br>· Heavy DIY (*eg*: weeding, lawn mowing, carpentry, digging) =0;<br>· Other exercises (*eg*: swimming, cycling, keep fit, bowling) =0;<br>· None of the above=1;<br>· Prefer not to answer=excluded	6164; 1011

Indicators of physical frailty were measured at both baseline and follow-up visits. In UK Biobank, each field had a unique Field ID, and the instance index was used to distinguish the data field which was gathered at a different time (0 for the baseline visit, and 2 for the imaging visit). Therefore, the UK Biobank Field IDs for calculating physical frailty at baseline were 2306-0.0, 2080-0.0, 924-0.0, 46-0.0, 47-0.0, 6164-0.0, and 1011-0.0; Field IDs for calculating physical frailty at follow-up visit were 2306-2.0, 2080-2.0, 924-2.0, 46-2.0, 47-2.0, 6164-2.0, and 1011-2.0.

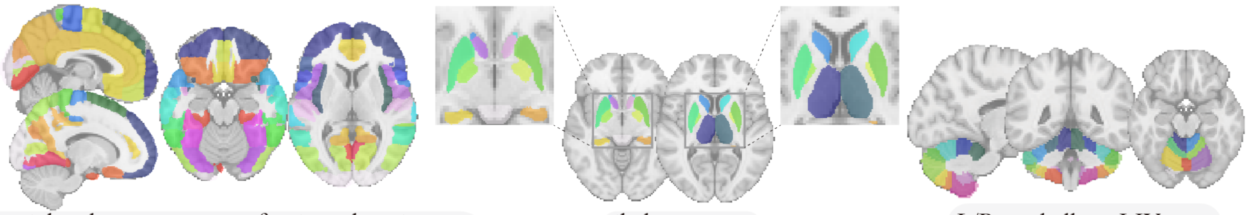

Harvard-Oxford cortical atlas (48 left+48 right)		Harvard-Oxford subcortical atlas (7 left+7 right+brain stem)	Diedrichsen cerebellar atlas (28 regions)
frontal_pole	front_med_cortex	thalamus	L/R.cerebellum_I-IV
insular_cortex	juxtapos_lobule_cortex	caudate	L/R.cerebellum_V
sup_front_gyrus	subcallosal_cortex	putamen	L/V/R.cerebellum_VI
mid_front_gyrus	paracing_gyrus	pallidum	L/V/R.cerebellum_crus_I
inf_front_gyrus_parstri	cing_gyrus_ant	hippocampus	L/V/R.cerebellum_crus_II
inf_front_gyrus_parsop	cing_gyrus_post	amygdala	L/V/R.cerebellum_VIIb
precentral_gyrus	precun_cortex	accumbens	L/V/R.cerebellum_VIIIa
temporal_pole	cuneal_cortex	brain_stem	L/V/R.cerebellum_VIIIb
sup_temp_gyrus_ant	front_orb_cortex		/V/RL.cerebellum_IX
sup_temp_gyrus_post	parahipp_gyrus_ant		L/V/R.cerebellum_X
mid_temp_gyrus_ant	parahipp_gyrus_post		
mid_temp_gyrus_post	lingual_gyrus		
mid_temp_gyrus_tempocc	temp_fusif_cortex_ant		
inf_temp_gyrus_ant	temp_fusif_cortex_post		
inf_temp_gyrus_post	temp_occ_fusif_cortex		
inf_temp_gyrus_tempocc	occ_fusif_gyrus		
postcent_gyrus	front_operc_cortex		
sup_parietal_lobule	cent_operc_cortex		
supramarg_gyrus_ant	parietal_operc_cortex		
supramarg_gyrus_post	planum_polare		
angular_gyrus	heschl_gyrus		
latocc_cortex_sup	planum_temporale		
latocc_cortex_inf	supracalc_cortex		
intracalc_cortex	occ_pole		

Figure S2. List of names for 139 brain regions. All brain MRI data were acquired on a 3T Siemens Skyra scanner using a standard 32-channel head coil. Of relevance to this study, T1-weighted MPRAGE were obtained in sagittal orientation using the following parameters: resolution: 1×1×1 mm, field-of-view (FOV): 208×256×256 matrix, duration: 5 minutes; T2-weighted FLAIR volumes were acquired in sagittal orientation using the following parameters: resolution: 1.05×1×1 mm, FOV: 192×256×256 matrix, duration: 6 minutes. The tissue-type segmentation was applied using FAST (FMRIB's Automated Segmentation Tool), and subcortical structures were modeled using FIRST (FMRIB's Integrated Registration and Segmentation Tool)[2]. The 139 brain regions include 96 cortical and 15 subcortical regions based on Harvard-Oxford atlas (https://fsl.fmrib.ox.ac.uk/fsl/fslwiki/Atlases), and 28 cerebellar regions based on Diedrichsen cerebellar atlas (http://www.diedrichsenlab.org/imaging/propatlas.htm). GMV segmentation based on FAST and FIRST is one of the most widely used methods to delineate gray matter structures from MR images. Among the multiple tools to segment brain structure in UK Biobank, we chose the current one because it has been widely used in other studies investigating the association between GMV and multiple behaviors (e.g., smoking[3], alcohol[4], obesity[5], diabetes[6], grip strength[7], and etc.), making our results amenable to replication. Additionally, segmentation based on FAST and FIRST generated 139 brain regions including cortical, subcortical, and cerebellar areas. In contrast, segmentation based on Freesurfer only includes cortical and subcortical regions but not cerebellar areas.

Table S2. Characteristic of participants used in the current study

	Cross-sectional analyses	Longitudinal analyses	Imaging analyses	Field ID
Total N	483,033	46,501	40,210	
Age (mean ± sd)<br>range	56.51±8.08<br>38~73	64.12±7.73<br>44~82	63.87±7.67<br>44~82	21003
Sex, N/%				31
Female	262,784/54.40	23,907/51.41	21,220/52.77	
Male	220,249/45.60	22,594/48.59	18,990/47.23	
Ethnicity, N/%				21000
White	457,357/94.68	45,030/96.84	38,935/96.83	
Non-White	25,676/5.32	1,471/3.16	1,275/3.17	
Body mass index (kg/m^2, mean ± sd)	27.40±4.77	26.58±4.45	26.48±4.37	21001
Waist-to-hip ratio (mean ± sd)	0.87±0.090	0.88±0.089	0.87±0.088	48; 49
Data acquisition date (range)	12/19/2006<br>10/01/2010	04/30/2014<br>03/13/2020	05/02/2014<br>03/13/2020	53
Townsend deprivation index (mean ± sd)	-1.34±3.07	-1.89±2.72	-1.90±2.72	189
Average total household income, N/%				738
Low (<£51,999)	308,637/63.90	29,551/63.55	25,394/63.15	
Middle (£52,000–£100,000)	85,435/17.69	9,658/20.77	8,454/21.02	
High (>£100,000)	22,757/4.71	3,020/6.49	2,694/6.70	
Unknown	66,204/13.71	4,272/9.19	3,668/9.12	
Education levels, N/%				6138
College/University	158,986/32.91	22,579/48.56	19,769/49.16	
Less than College	324,047/67.09	23,922/51.44	20,441/50.84	
Smoking status, N/%				20116
Never	263,871/54.63	28,838/62.02	25,177/62.61	
Ever	16,7331/34.64	15,897/34.19	13,550/33.70	
Current	50,259/10.40	1,627/3.50	1,366/3.40	
Unknown	1,572/0.33	139/0.30	117/0.29	
Alcohol intake frequency, N/%				1558
Daily or almost daily	99,181/20.53	8,009/17.22	6,833/16.99	
Three or four times a week	112,478/23.29	13,046/28.06	11,327/28.17	
Once or twice a week	124,983/25.87	12,207/26.25	10,633/26.44	
One to three times a month	53,918/11.16	5,359/11.52	4,646/11.55	
Special occasions only	54,773/11.34	4,767/10.25	4,137/10.29	
Never	37,379/7.74	3,103/6.67	2,626/6.53	
Unknown	321/0.07	10/0.02	8/0.02	
Frailty severity, N/%				
0	267091/55.29	26,328/56.62	22,991/57.18	
1	148536/30.75	15,193/32.67	13,059/32.48	
2	47370/9.81	3,921/8.43	3,285/8.17	
3	14918/3.09	849/1.83	700/1.74	
4	4513/0.93	197/0.42	156/0.39	
5	605/0.13	22/0.05	19/0.05	

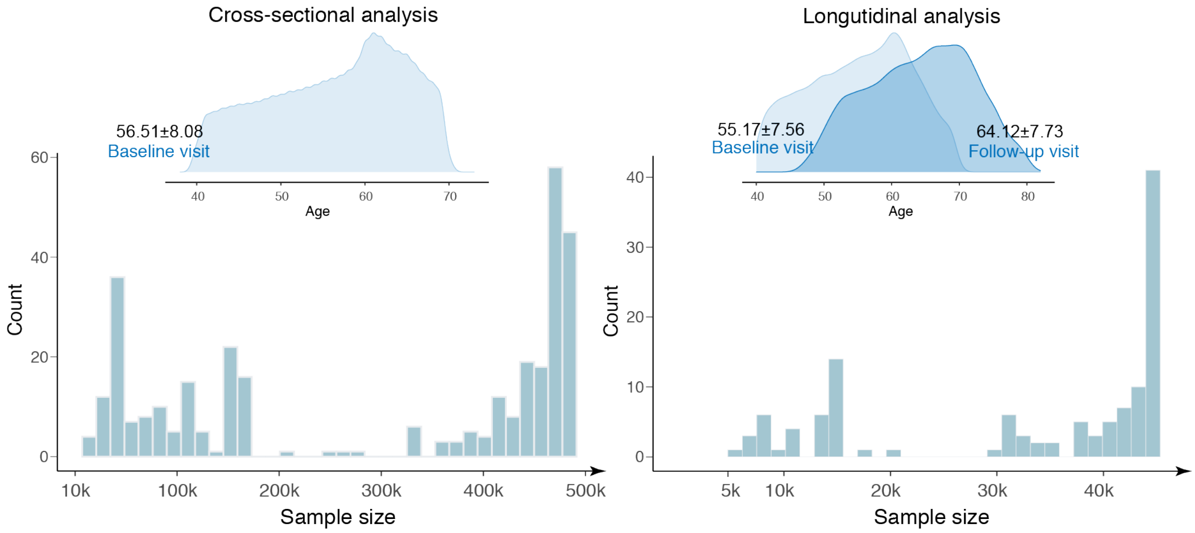

Figure S3. Distribution of age and sample sizes of participants used in cross-sectional and longitudinal analyses.

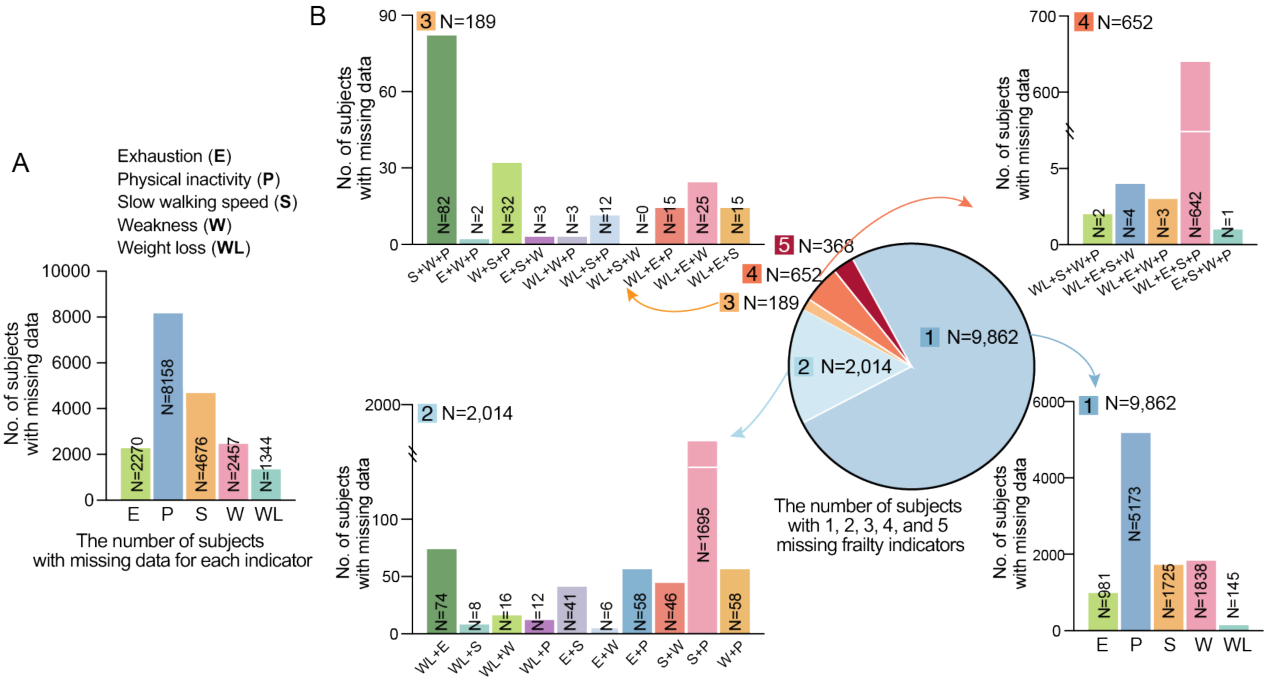

Figure S4. Missingness of frailty indicators in UK Biobank. A total of 13,085 participants were excluded from our analysis due to missing data on at least one of the five frailty indicators (as shown in Figure S1). (**A**) The number of participants with missing data for each indicator was: N=2270 (exhaustion), N=8158 (physical inactivity), N=4676 (slow walking speed), N=2457 (weakness), and N=1344 (weight loss). Among the five frailty indicators, 'Physical inactivity' had the highest number of missing participants. This can be partially attributed to the fact that the derivation of 'physical inactivity' was based on two fields (physical type and physical frequency), while the other indicators were derived based on only one field. 'Weight loss' had the least number of missing participants. (**B**) The number of participants with one (N=9862), two (N=2014), three (N=189), four (N=652), and five (N=368) missing frailty indicators, and the detailed missingness for each condition. 75.37% (9862/13,085) of all missing participants had only one missing indicator of frailty. For participants with only one missing indicator, 'Physical inactivity' was the most common reason of missingness, and the number of missing participants in each of the other four indicators was small. For participants with only two missing indicators, 'Slow walking speed' + 'Physical inactivity' was the most common combination.

Table S3. Characteristic of participants with all frailty indicators (the complete-case sample) and those missing at least one frailty indicator

	Complete-case sample	Missing data on frailty	P for difference
Total N	483,033	13,085	
Age (mean ± sd)<br>range	56.51±8.08<br>38~73	56.55±8.31<br>37~70	0.54
Sex, N/%			0.0004
Female	262,784/54.40	7,324/55.97	
Male	220,249/45.60	5,761/44.03	
Ethnicity, N/%			P<0.001
White	457,357/94.68	10,098/77.17	
Non-White	25,676/5.32	2,987/22.83	
Body mass index (kg/m^2, mean ± sd)	27.40±4.77	28.52±5.88	P<0.001
Waist-to-hip ratio (mean ± sd)	0.87±0.090	0.88±0.094	P<0.001
Data acquisition date (range)	12/19/2006<br>10/01/2010	03/13/2006<br>09/22/2010	NA
Townsend deprivation index (mean ± sd)	-1.34±3.07	-0.002±3.55	P<0.001
Average total household income, N/%			P<0.001
Low (<£51,999)	308,637/63.90	4,855/37.10	
Middle (£52,000–£100,000)	85,435/17.69	513/3.92	
High (>£100,000)	22,757/4.71	107/0.82	
Unknown	66,204/13.71	7,610/58.16	
Education levels, N/%			P<0.001
College/University	158,986/32.91	1,684/12.87	
Less than College	324,047/67.09	6,060/46.31	
Unknown	0/0	5,341/40.82	
Smoking status, N/%			P<0.001
Never	263,871/54.63	6,241/47.70	
Ever	16,7331/34.64	3,714/28.38	
Current	50,259/10.40	1,889/14.44	
Unknown	1,572/0.33	1,241/9.48	
Alcohol intake frequency, N/%			P<0.001
Daily or almost daily	99,181/20.53	1,777/13.58	
Three or four times a week	112,478/23.29	1,923/14.70	
Once or twice a week	124,983/25.87	2,683/20.50	
One to three times a month	53,918/11.16	1,226/9.37	
Special occasions only	54,773/11.34	2,135/16.32	
Never	37,379/7.74	2,196/16.78	
Unknown	321/0.07	1,145/8.76	

A total of 13,085 participants were excluded from our analysis due to having missing data on at least one of the five frailty indicators. This table shows the characteristic of the complete-case sample (with complete data on frailty and basic covariates) and those missing at least one frailty indicator. Results indicated that the excluded participants were more likely to be females, non-White, and materially deprived, had lower education levels and family income, and higher body-mass index and waist-to-hip ratio. They were also more likely to smoke. Notably, participants having missing data on one or more indicators of frailty were also more likely to have missing data on covariates, compared with the complete-case sample. The mean values of body-mass index, waist-to-hip ratio, and Townsend deprivation index for the excluded participants were calculated based on complete data.

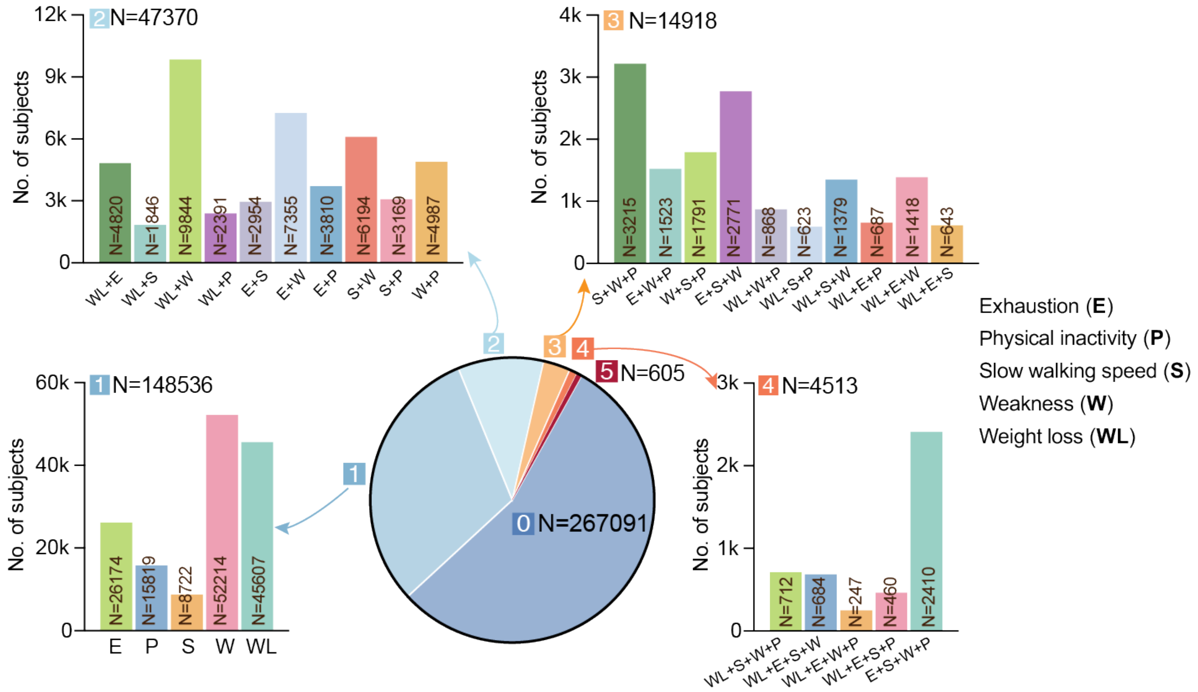

Figure S5. The number of participants with 0, 1, 2, 3, 4, and 5 frailty indicators was N=267,091, N=148,536, N=47,370, N=14,918, N=4,513, N=1344, and N=605, respectively. The barplot shows the number of subjects with different combinations of frailty indicators.

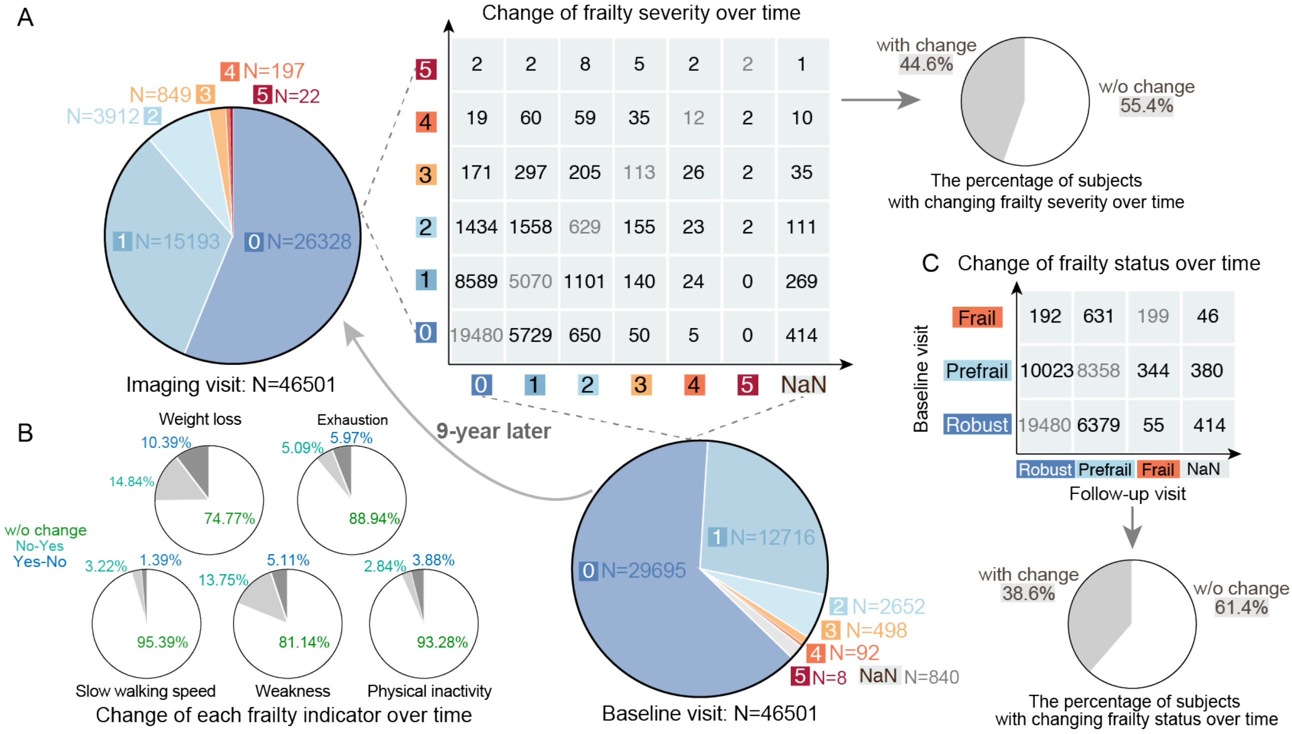

Figure S6. Fluctuation of severity of physical frailty over time (9-year). (**A**) We included up to 46,501 participants for longitudinal association analyses. The number of participants with 0, 1, 2, 3, 4, and 5 frailty indicators at baseline and 9-year follow-up is shown in the pie chart. The frailty severity at baseline was significantly correlated with that at follow-up (r=0.36, P<10^{-10}). The cell plot shows how the frailty severity fluctuates over time. Overall, a substantial proportion (44.6%, 20355/45661) of UK Biobank participants experienced at least one transition over time, which included both worsening and improvement in frailty severity. (**B**) Change of each frailty indicator over time. Response to each of the five frailty indicators was dichotomized into 'Yes' (which means the indicator presents) or 'No' (which means the indicator does not present). Among all five indicators, weight loss (25.23%) showed the highest rate of change over time, followed by weakness (18.86%), and exhaustion (11.06%). Physical inactivity (6.72%) and slow walking speed (4.61%) showed a low percentage of change. (**C**) Participants were classified as frail if they fulfilled three or more of the five criteria, prefrail if they fulfilled one or two criteria, and robust if they did not fulfill any criteria [8-11]. The percentage of participants showing changes in frailty status (robust, prefrail, and frail) was 38.6% (17624/45661).

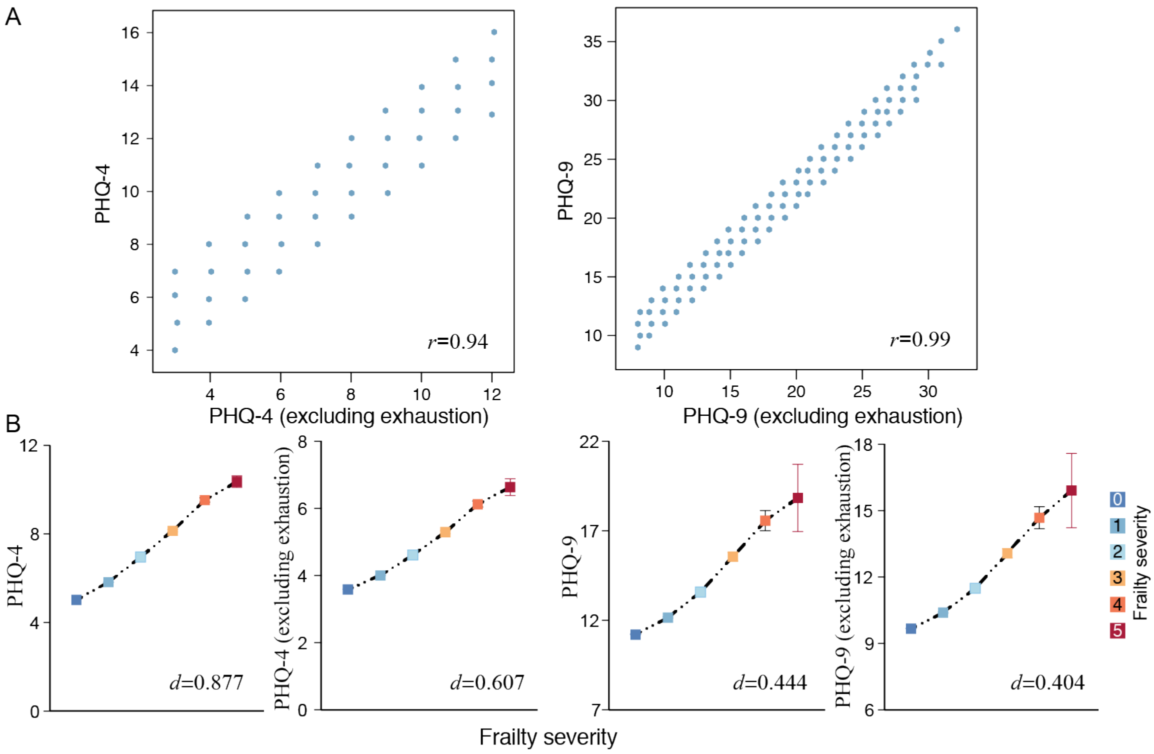

Figure S7. Association between physical frailty and depressive symptoms as measured by patient health questionnaire (PHQ). The four-item PHQ was used to assess the depressive symptoms at the UK Biobank assessment center and then repeated at the neuroimaging visit. A detailed and comprehensive mental health questionnaire was administered online to assess self-reported symptoms of mental disorders, where the nine-item PHQ was used to assess depressive symptoms. The PHQ-4 includes the item 'exhaustion' (*Field ID*: 2080), which is a component of physical frailty; the PHQ-9 includes a similar component (*Field ID*: 20519). (**A**) The PHQ scores demonstrated a high correlation between the cases with or without including the 'exhaustion' item (*r*[PHQ-4]=0.94, *r*[PHQ-9]=0.99). (**B**) The association between physical frailty and PHQ-4 was attenuated when excluding the 'exhaustion' item from the calculation of PHQ-4 scores (from d=0.877 to d=0.607). For PHQ-9, excluding the 'exhaustion' item had minimal effects on its association with physical frailty (from d=0.444 to d=0.404).

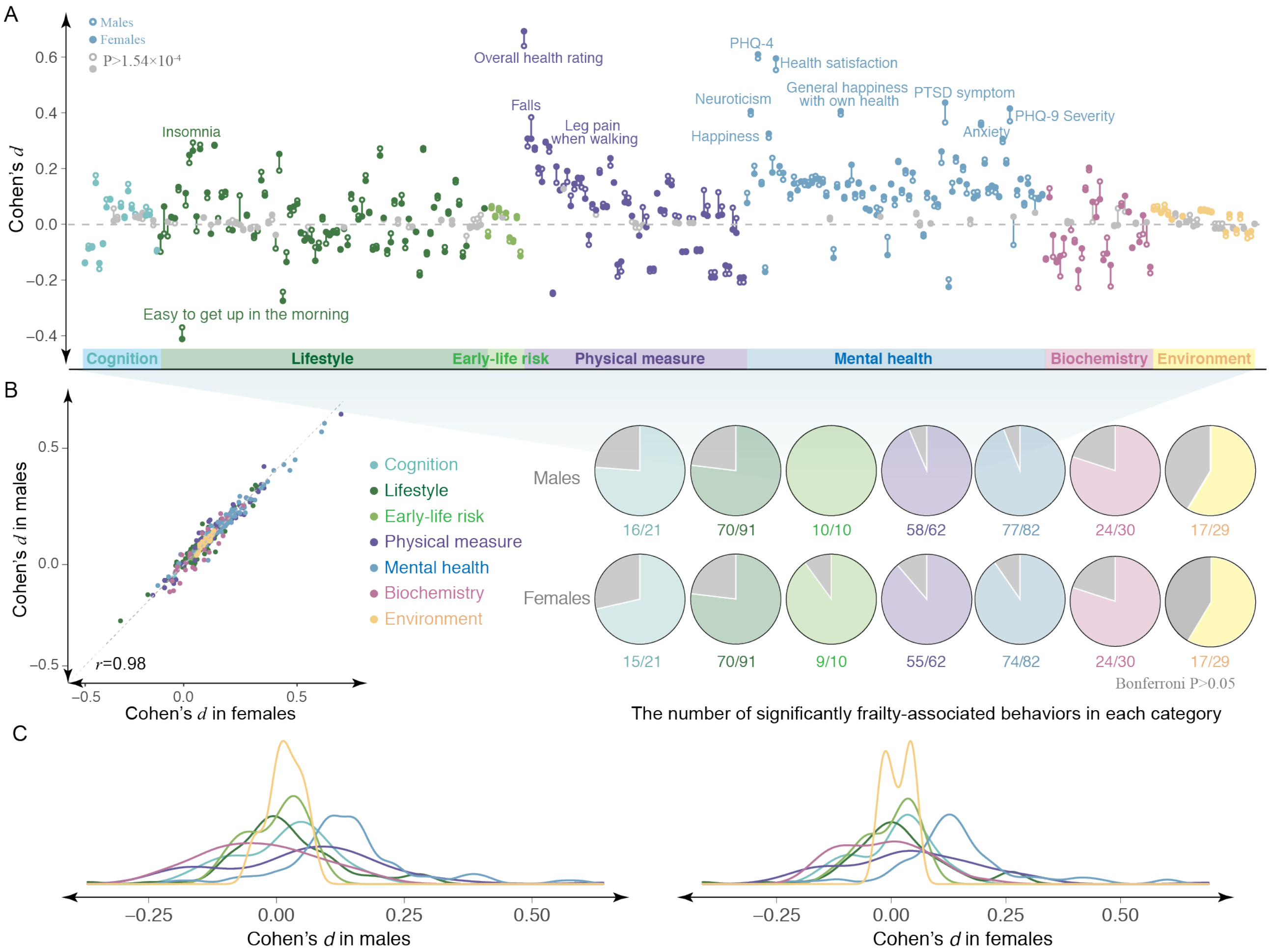

Figure S8. Associations between frailty and health-related measures stratified by gender. (**A**) A respective of 272 and 264 health-related measures of all 325 examined showed significant associations with physical frailty for males and females. (**B**) Association patterns were highly similar between males and females (*r*=0.97). (**C**) Distributions of effect sizes of associations for males and females. Among all seven categories, mental health measures showed the strongest associations with frailty, regardless of gender. $P<1.54\times10^{-4}$, the Bonferroni corrected level of association.

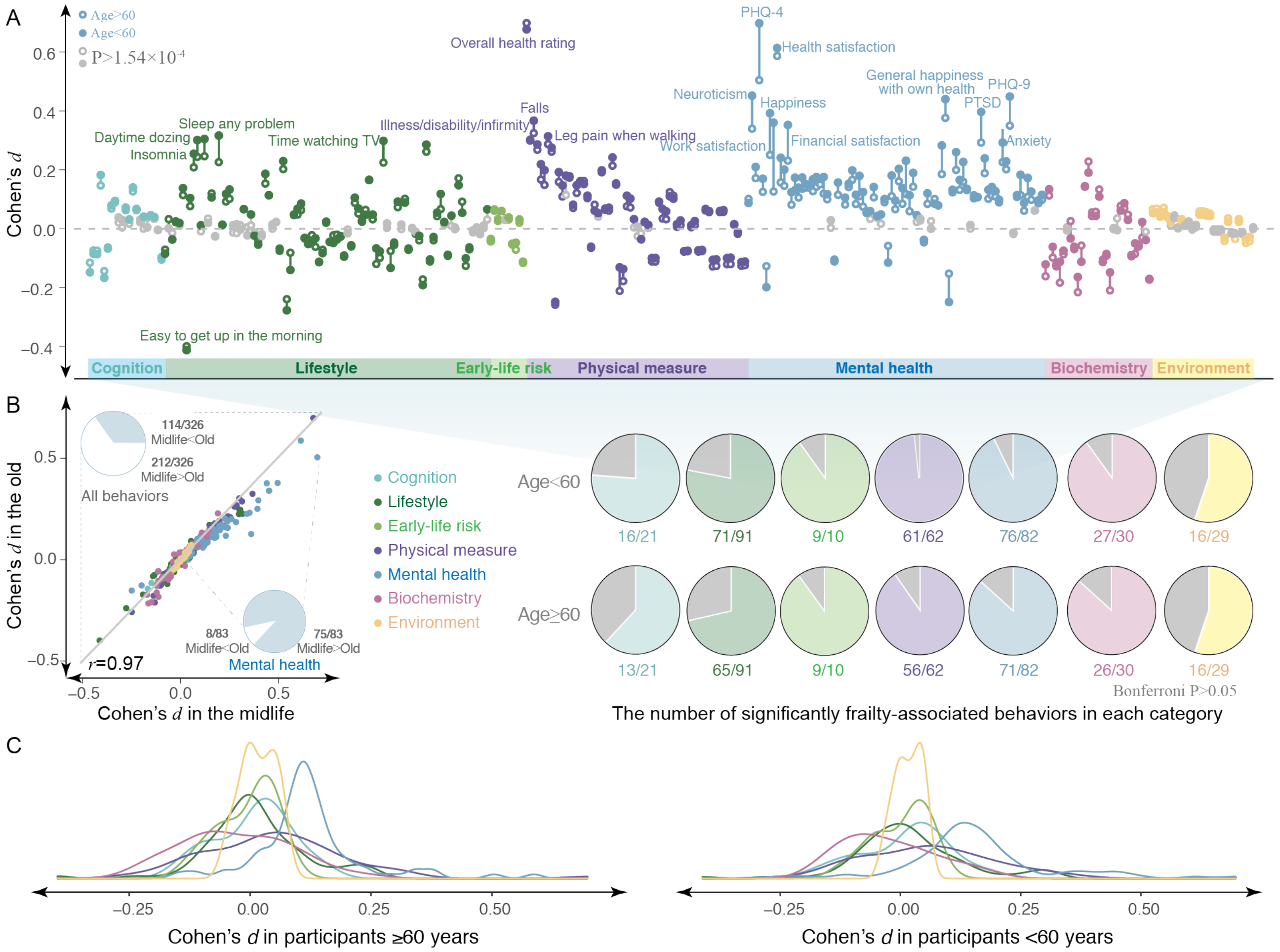

Figure S9. Associations between frailty and health-related measures stratified by age. To ensure an approximately comparable number of samples between subgroups, we used 60 years as the cutoff, and finally derived the midlife group (45~60 years old) and old group (>60 years old). Participants aged 45 and younger, which only accounted for a small portion of the entire samples, were excluded from the sensitivity analysis. (**A**) A respective of 276 and 256 health-related measures of all 325 examined showed significant associations with physical frailty for participants in the midlife group and old group. (**B**) Association patterns were highly similar between the two age groups (r=0.97). Notably, people aged 45—60 years showed larger effect sizes, and larger proportion of significant associations (76/82 in midlife group, and 71/82 in old group) in mental health measures than their older counterparts. (**C**) Distributions of effect sizes of associations for these two age groups. Among all seven categories, mental health measures showed the strongest associations with frailty, regardless of age group. $P<1.54\times10^{-4}$, the Bonferroni corrected level of association.

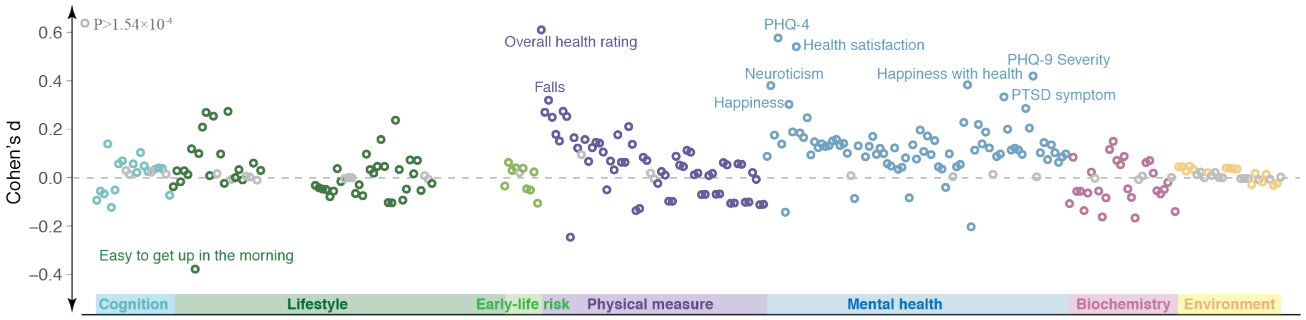

Figure S10. Associations between frailty and health-related measures after additionally controlling for confounders of family income, smoking status, and alcohol consumption frequency. Information about family income was not available for many participants. The average total household income was self-reported and categorized as low (<£51,999), middle (£52,000–£100,000), and high (>£10,000). Smoking status was self-reported including never, ever, and current. Alcohol intake frequency includes daily or almost daily, three or four times a week, once or twice a week, one to three times a month, special occasions only, and never. Overall, the number of participants meeting the inclusion criteria for this analysis was 11,836–416,829 per specific health measure.

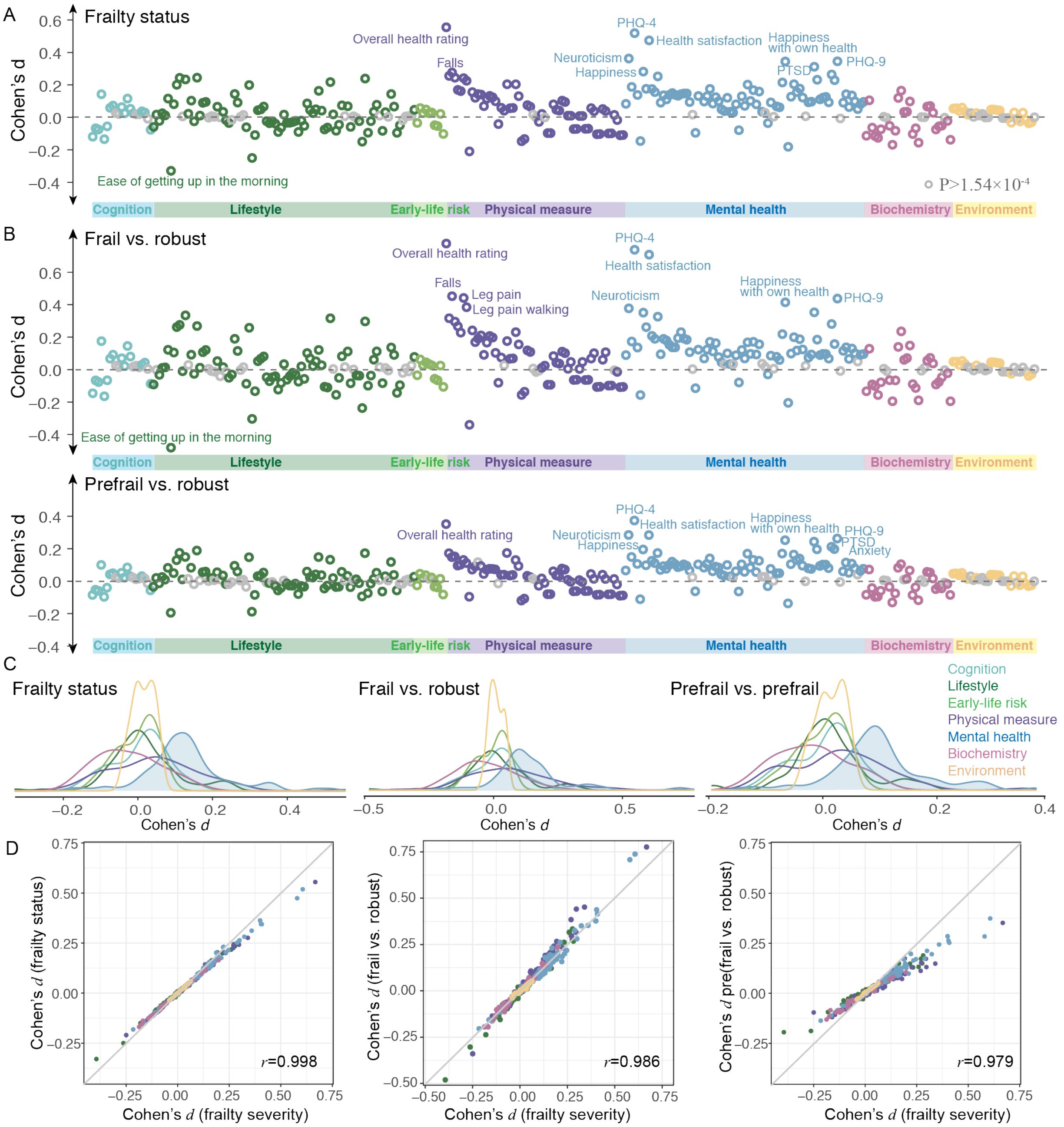

Figure S11. Sensitivity analyses of associations between physical frailty and health-related outcomes. Participants were classified as frail if they fulfilled three or more of the five criteria, prefrail if they fulfilled one or two criteria, and robust if they did not fulfill any criteria [8-10]. (**A**) Associations between physical frailty as assessed by frailty status (robust, prefrail, and frail) and health-related outcomes. $P<1.54\times10^{-4}$, the Bonferroni corrected level of association. (**B**) Associations between physical frailty and health-related outcomes when treating frailty as a categorical variable and using robust participants as the reference group. (**C**) Distribution of effect sizes. Among all seven categories, mental health showed the strongest association with frailty. (**D**) Association patterns were highly similar between the cases treating frailty as a continuous variable (frailty severity) or categorical variable (*r*[frailty severity-frailty status]=0.998, *r*[frailty severity-frail vs. robust]=0.986, *r*[frailty severity-frail vs. prefrail]=0.979).

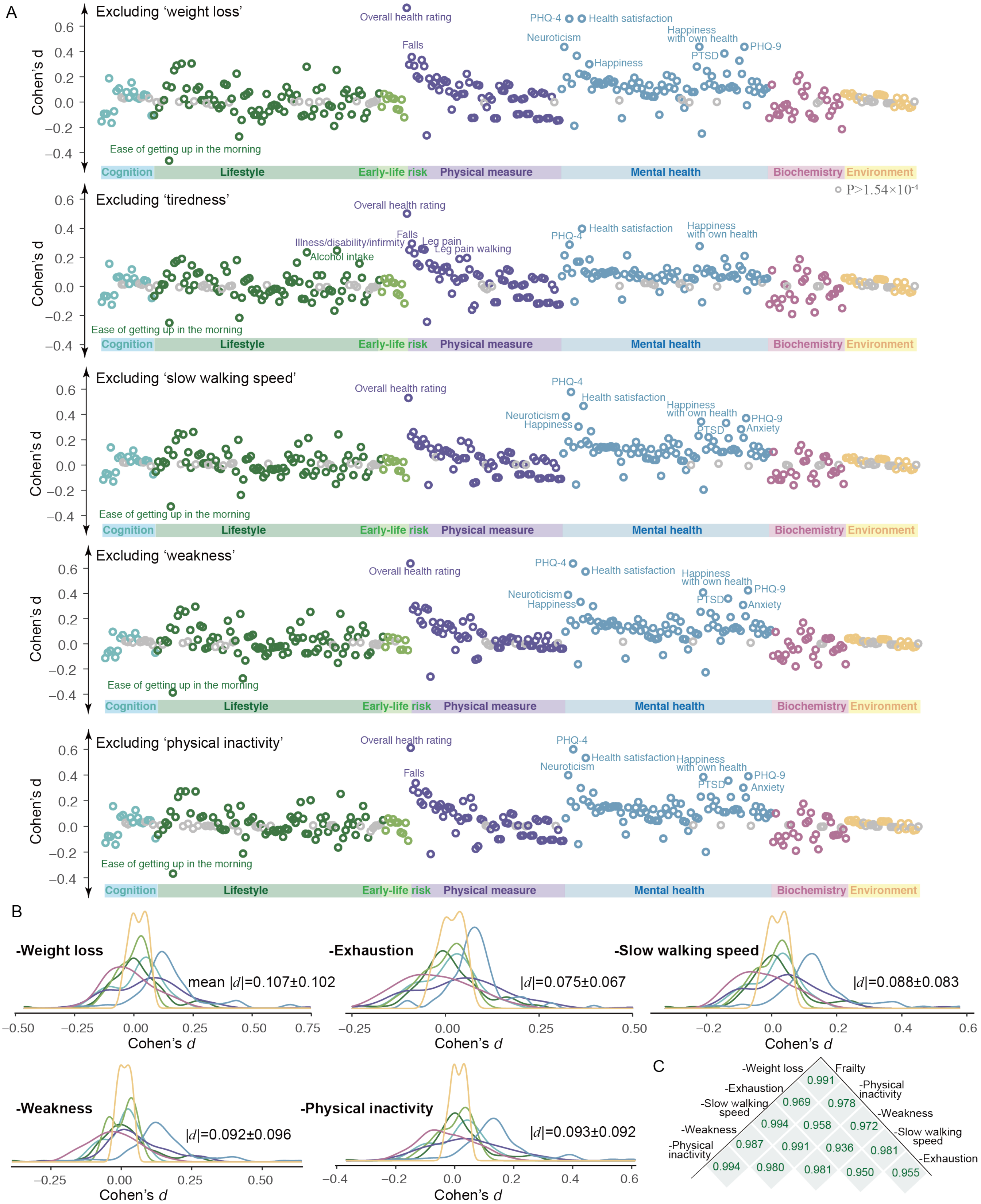

Figure S12. To investigate whether associations between physical frailty and health-related outcomes were merely driven by any single indicator of frailty, we excluded an indicator from the 5-indicator frailty phenotype and then fit association models. (**A**) Associations between 4-indicator physical frailty and health-related outcomes. Bonferroni correction at a significance level of *P*<1.54×10^{-4} was used to account for similar tests across 325 outcomes. (**B**) Distribution of effect sizes. The mean absolute effect size across 325 outcomes was |d|=0.107±0.102 (excluding weight loss), |d|=0.075±0.067 (excluding exhaustion), |d|=0.088±0.083 (excluding slow walking speed), |d|=0.092±0.096 (excluding weakness), and |d|=0.093±0.092 (excluding physical inactivity). (**C**)

Correlation of association patterns between different combinations of frailty indicators (the similarity ranged from *r*[-weakness vs. -exhaustion]=0.0.936 to *r*[-physical inactivity vs. -slow walking speed]=0.994). Association patterns were highly similar between cases excluding any single indicator from the frailty phenotype. Moreover, each of these association patterns was also highly correlated with that derived using 5-indicator frailty severity phenotype. This result suggests that the association between frailty severity and health-related outcomes was not driven by any single indicator. Among all conditions, excluding 'exhaustion' had the smallest effect sizes (mean $|d|=0.075\pm0.067$), which may suggest that this indicator was more associated with these health outcomes. In contrast, excluding 'weight loss' showed the largest effect size among all five conditions (mean $|d|=0.107\pm0.102$), indicating that this indicator may less relate to these health outcomes. This can be also attributed to the fact that we used self-reported weight loss, rather than the originally defined 'unintentionally loss of >10 pounds'[12]. Including some people who lost weight deliberately may underestimate the associations [9].

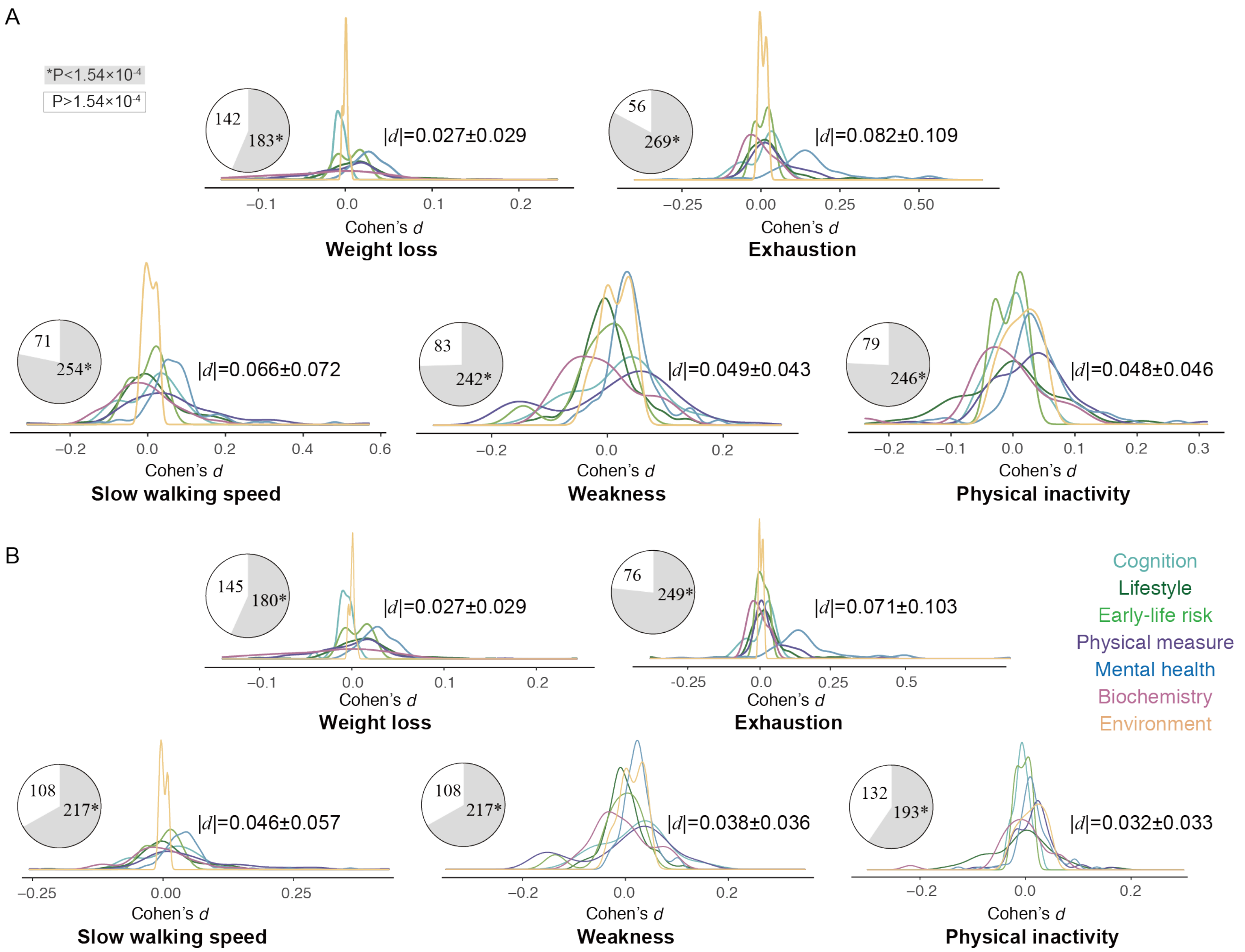

Figure S13. Associations between each indicator of physical frailty and health-related outcomes while controlling for numerous confounders. (**A**) We fit separate models to examine the association between each individual indicator of physical frailty and 325 health-related outcomes. The density plots show the distributions of effect sizes of associations. Among all five frailty indicators, 'Exhaustion' showed the strongest mean association with health-related outcomes, followed by slow walking speed; weight loss showed the lowest mean effect size. The pie charts show the number of significant/insignificant associations (P<1.54×10^{-4}, the Bonferroni corrected level of association). The number of health-related outcomes that were independently associated with each frailty indicator was n=183 (weight loss), n=269 (exhaustion), n=254 (slow walking speed), n=242 (weakness), and n=246 (physical inactivity). (**B**) We investigated the independent association of each individual frailty indicator with health-related outcomes while including mutual adjustment of the other four indicators of frailty [8]. The associations between each frailty indicator and health-related outcomes were attenuated following mutual adjustment of other indicators of frailty. Among all five indicators, 'Exhaustion' showed the strongest mean association with health-related outcomes independently of the other indicators; 'Weight loss' showed the lowest independent association. The number of health-related outcomes that were independently associated with each frailty indicator was n=180 (weight loss), n=249 (exhaustion), n=217 (slow walking speed), n=217 (weakness), and n=193 (physical inactivity). The magnitude of associations related to individual indicators was much lower than that related to the frailty severity (mean |d|=0.10 across 325 outcomes, Figure 2B), indicating the significance of using the frailty phenotype to characterize health-related outcomes. Overall, these results suggest that each indicator of physical frailty demonstrated an independent association with health-related outcomes, with 'Exhaustion' and 'Slow walking speed' being the most significant indicators and 'Weight loss' being the least significant one.

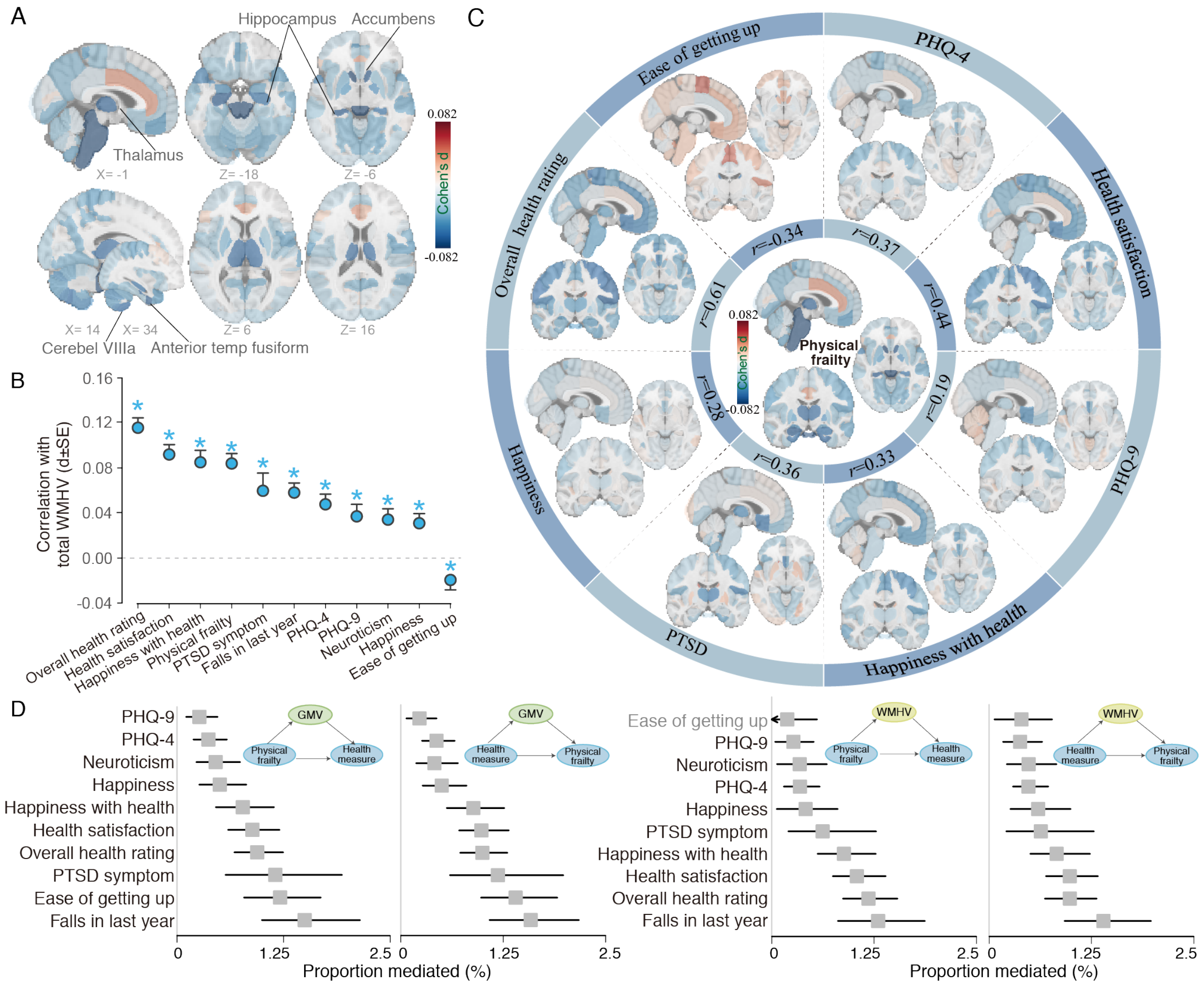

Figure S14. Brain structures were correlated with physical frailty and mediated the relationship between health outcomes and physical frailty. (**A**) The regional analysis revealed widespread significant associations between physical frailty and GMV while adjusting for potential confounders. (**B**) Both physical frailty and its top ten correlated health measures showed significant correlations with tWMH (P_{FDR}<0.05). (**C**) The association map between GMV and frailty was significantly similar to eight of the top ten frailty-correlated health measures (P_{FDR}<0.05). (**D**) The mean GMV of brain regions that were significantly correlated with both frailty and the health measure significantly and partially mediated the effect of frailty on all top 10 frailty-correlated health outcomes, or the effect of health outcomes on frailty, with the proportion of mediated variance ranging from 0.23% to 1.59% (P_{FDR}<0.05). The mediation analyses also revealed a significant indirect effect of tWMH on the association between frailty and health outcomes in both directions except for "Ease of getting up". The explained effect of tWMH ranged from 0.27% to 1.40%. GMV, grey matter volume; tWMH: total white matter hyperintensity.

Table S4. Regional associations between GMV and physical frailty

Brain regions	T-value	P_{FDR}	β	95% CI Lower	95% CI Upper	Cohen's d	N
L. accumbens	-8.68	5.88×10^{-16}	-0.041	-0.05	-0.032	-0.082	40,203
L.thalamus	-8.03	6.75×10^{-14}	-0.035	-0.043	-0.026	-0.07	40,158
R. accumbens	-7.98	7.21×10^{-14}	-0.037	-0.046	-0.028	-0.074	40,201
R.thalamus	-7.93	7.54×10^{-14}	-0.034	-0.043	-0.026	-0.069	40,157
brain_stem	-7.89	8.31×10^{-14}	-0.04	-0.05	-0.03	-0.081	40,158
L.hippocampus	-7.85	9.82×10^{-14}	-0.036	-0.045	-0.027	-0.072	40,183
L.temp_fusif_cortex_ant	-7.8	1.29×10^{-13}	-0.038	-0.048	-0.029	-0.077	40,199
R.temp_fusif_cortex_ant	-7.44	1.73×10^{-12}	-0.037	-0.047	-0.027	-0.074	40,196
R.cerebellum_VIIIa	-7.03	3.23×10^{-11}	-0.033	-0.042	-0.024	-0.066	40,200
R.parahipp_gyrus_ant	-6.8	1.49×10^{-10}	-0.034	-0.044	-0.024	-0.068	40,187
L.cerebellum_VIIIa	-6.78	1.49×10^{-10}	-0.032	-0.041	-0.023	-0.064	40,199
R.hippocampus	-6.51	9.06×10^{-10}	-0.03	-0.04	-0.021	-0.061	40,174
L.cerebellum_VIIIb	-6.3	3.13×10^{-09}	-0.03	-0.04	-0.021	-0.061	40,202
L.parahipp_gyrus_ant	-6.29	3.26×10^{-09}	-0.031	-0.041	-0.022	-0.063	40,197
L.cerebellum_VIIb	-6.1	9.75×10^{-09}	-0.029	-0.039	-0.02	-0.059	40,196
R.cerebellum_VIIb	-6.03	1.45×10^{-08}	-0.029	-0.039	-0.02	-0.058	40,191
R.cerebellum_VIIIb	-5.95	2.18×10^{-08}	-0.028	-0.037	-0.019	-0.056	40,205
L.temporal_pole	-5.81	4.94×10^{-08}	-0.028	-0.038	-0.019	-0.057	40,185
R.cerebellum_crus_I	-5.79	5.14×10^{-08}	-0.027	-0.037	-0.018	-0.055	40,196
R.pallidum	-5.59	1.58×10^{-07}	-0.027	-0.037	-0.018	-0.055	40,145
L.postcent_gyrus	-5.41	4.21×10^{-07}	-0.025	-0.034	-0.016	-0.049	40,182
L.cerebellum_crus_II	-5.39	4.52×10^{-07}	-0.026	-0.035	-0.016	-0.051	40,194
R.postcent_gyrus	-5.25	9.00×10^{-07}	-0.024	-0.033	-0.015	-0.048	40,190
L.cerebellum_crus_I	-5.21	1.12×10^{-06}	-0.024	-0.034	-0.015	-0.049	40,199
L.amygdala	-5.12	1.75×10^{-06}	-0.026	-0.036	-0.016	-0.053	40,183
L.front_med_cortex	-5.1	1.86×10^{-06}	-0.025	-0.035	-0.015	-0.05	40,193
L.pallidum	-4.96	3.58×10^{-06}	-0.025	-0.035	-0.015	-0.05	40,124
R.cent_operc_cortex	-4.89	5.09×10^{-06}	-0.023	-0.033	-0.014	-0.046	40,196
R.cerebellum_crus_II	-4.87	5.39×10^{-06}	-0.023	-0.032	-0.014	-0.046	40,188
R.temporal_pole	-4.86	5.39×10^{-06}	-0.024	-0.033	-0.014	-0.047	40,185
L.cerebellum_VI	-4.6	1.90×10^{-05}	-0.022	-0.031	-0.012	-0.043	40,199
R.sup_temp_gyrus_post	-4.59	1.93×10^{-05}	-0.022	-0.032	-0.013	-0.045	40,195
R.cerebellum_VI	-4.58	1.94×10^{-05}	-0.022	-0.031	-0.012	-0.043	40,195
R.cerebellum_IX	-4.52	2.57×10^{-05}	-0.022	-0.031	-0.012	-0.043	40,206
R.mid_temp_gyrus_post	-4.46	3.20×10^{-05}	-0.022	-0.031	-0.012	-0.043	40,197
R.precentral_gyrus	-4.45	3.25×10^{-05}	-0.021	-0.03	-0.012	-0.041	40,183
L.cerebellum_IX	-4.45	3.30×10^{-05}	-0.021	-0.031	-0.012	-0.043	40,206
R.occ_fusif_gyrus	-4.39	4.07×10^{-05}	-0.021	-0.031	-0.012	-0.043	40,199
R.precun_cortex	-4.3	6.09×10^{-05}	-0.02	-0.03	-0.011	-0.041	40,185
L.putamen	-4.29	6.29×10^{-05}	-0.02	-0.029	-0.011	-0.04	40,166
R.amygdala	-4.17	1.03×10^{-04}	-0.022	-0.032	-0.011	-0.043	40,192
R.angular_gyrus	-4.14	1.16×10^{-04}	-0.021	-0.031	-0.011	-0.042	40,203
R.sup_parietal_lobule	-4.09	1.41×10^{-04}	-0.02	-0.03	-0.011	-0.041	40,203
R.lingual_gyrus	-4.02	1.86×10^{-04}	-0.02	-0.03	-0.01	-0.04	40,182
L.parahipp_gyrus_post	-4	1.99×10^{-04}	-0.019	-0.028	-0.01	-0.038	40,186
L.lingual_gyrus	-3.99	2.00×10^{-04}	-0.02	-0.03	-0.01	-0.04	40,187
L.occ_fusif_gyrus	-3.86	3.30×10^{-04}	-0.019	-0.029	-0.009	-0.038	40,198
L.precun_cortex	-3.85	3.49×10^{-04}	-0.019	-0.028	-0.009	-0.037	40,192
L.mid_temp_gyrus_ant	-3.78	4.54×10^{-04}	-0.019	-0.029	-0.009	-0.038	40,200
R.front_med_cortex	-3.7	5.98×10^{-04}	-0.018	-0.028	-0.009	-0.036	40,197
L.cerebellum_X	-3.7	5.90×10^{-04}	-0.018	-0.028	-0.009	-0.036	40,192
L.precentral_gyrus	-3.65	7.13×10^{-04}	-0.017	-0.026	-0.008	-0.034	40,188
V_cerebellum_X	-3.62	7.64×10^{-04}	-0.017	-0.026	-0.008	-0.033	40,204
V_cerebellum_VI	-3.61	8.04×10^{-04}	-0.017	-0.026	-0.008	-0.034	40,199

R.putamen	-3.52	1.08×10^{-03}	-0.017	-0.026	-0.007	-0.033	40,156
R.inf_temp_gyrus_tempocc	-3.51	1.09×10^{-03}	-0.018	-0.028	-0.008	-0.036	40,203
L.cing_gyrus_ant	3.5	1.12×10^{-03}	0.018	0.008	0.028	0.036	40,177
V_cerebellum_crus_II	-3.45	1.32×10^{-03}	-0.017	-0.027	-0.007	-0.034	40,202
L.inf_temp_gyrus_ant	-3.44	1.38×10^{-03}	-0.017	-0.027	-0.007	-0.035	40,202
R.heschl_gyrus	-3.4	1.56×10^{-03}	-0.016	-0.025	-0.007	-0.032	40,196
L.cerebellum_V	-3.36	1.79×10^{-03}	-0.016	-0.026	-0.007	-0.033	40,193
R.temp_occ_fusif_cortex	-3.34	1.90×10^{-03}	-0.017	-0.027	-0.007	-0.034	40,194
L.front_orb_cortex	-3.27	2.40×10^{-03}	-0.015	-0.025	-0.006	-0.031	40,186
L.insular_cortex	-3.2	3.02×10^{-03}	-0.015	-0.025	-0.006	-0.031	40,164
L.mid_temp_gyrus_post	-3.19	3.02×10^{-03}	-0.016	-0.025	-0.006	-0.031	40,191
R.insular_cortex	-3.15	3.45×10^{-03}	-0.015	-0.025	-0.006	-0.031	40,162
R.sup_temp_gyrus_ant	-3.09	4.05×10^{-03}	-0.016	-0.025	-0.006	-0.031	40,201
R.paracing_gyrus	-3.09	4.05×10^{-03}	-0.014	-0.023	-0.005	-0.029	40,190
L.temp_occ_fusif_cortex	-3.09	4.07×10^{-03}	-0.016	-0.026	-0.006	-0.031	40,199
L.subcallosal_cortex	-3.07	4.26×10^{-03}	-0.015	-0.025	-0.005	-0.03	40,180
R.parahipp_gyrus_post	-3.06	4.28×10^{-03}	-0.015	-0.024	-0.005	-0.029	40,193
L.cent_operc_cortex	-2.94	6.26×10^{-03}	-0.014	-0.023	-0.005	-0.028	40,195
L.supramarg_gyrus_post	-2.9	7.13×10^{-03}	-0.015	-0.025	-0.005	-0.029	40,198
R.inf_temp_gyrus_ant	-2.87	7.64×10^{-03}	-0.015	-0.024	-0.005	-0.029	40,202
L.paracing_gyrus	-2.84	8.24×10^{-03}	-0.013	-0.023	-0.004	-0.027	40,189

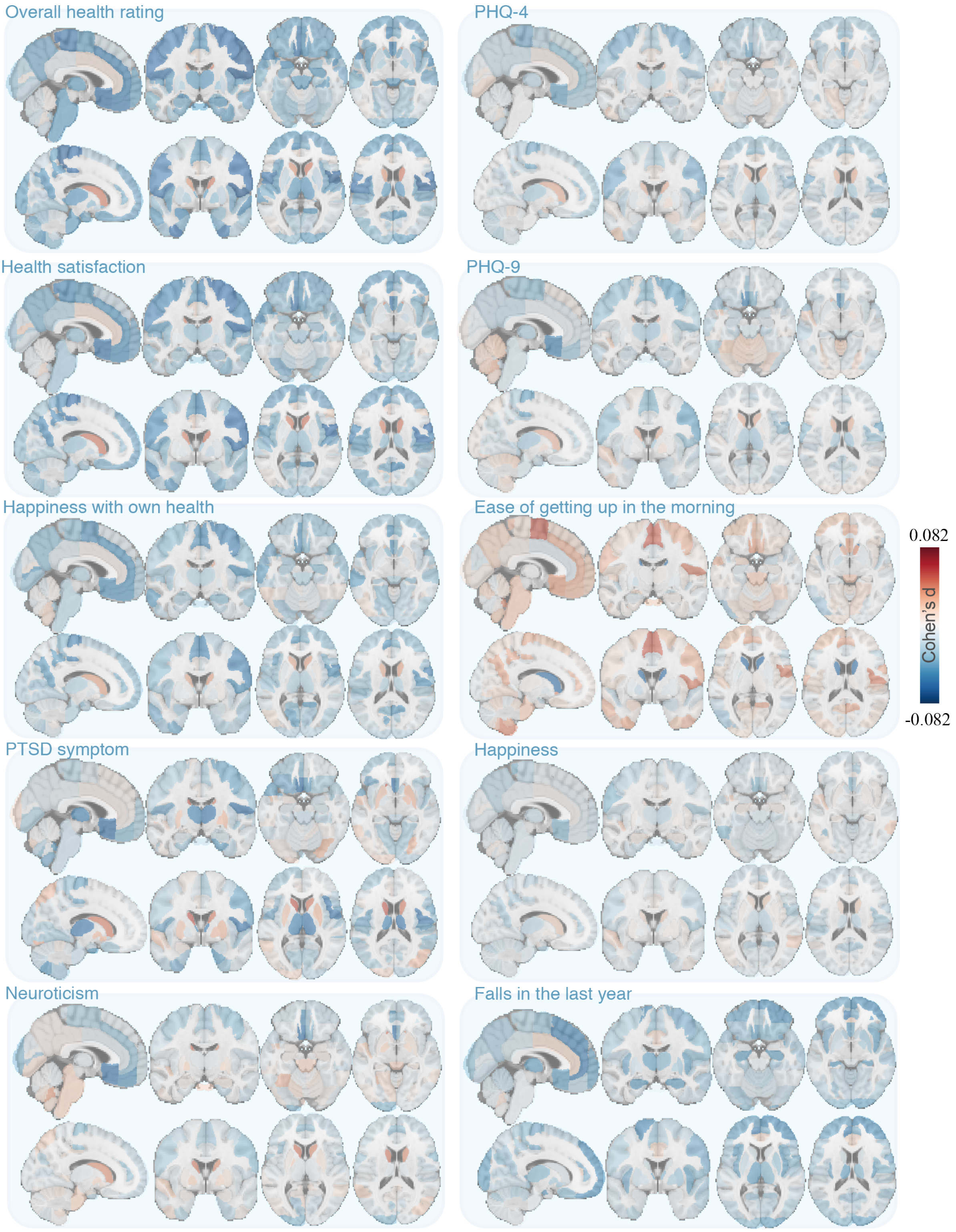

Figure S15. Regional distribution of associations between grey matter volume and the top ten frailty-correlated health measures. The similarity of association map between frailty and these health measures were: overall health rating (r=0.61, P=9.74×10^{-15}), PHQ-4 (r=0.37, P=2.06×10^{-5}), health satisfaction (r=0.44, P=3.22×10^{-7}), happiness with own health (r=0.61, P=9.74×10^{-15}), PHQ-9 (r=0.33, P=1.17×10^{-4}), neuroticism (r=-0.004, P=0.97), ease of getting up in the morning (r=-0.34, P=7.26×10^{-5}), PTSD (r=0.36, P=2.54×10^{-5}), falls (r=0.10, P=0.25), and general happiness (r=0.28, P=1.30×10^{-3}). All P-values were FDR corrected.

Table S5. Associations between white matter hyperintensities and health-related measures

Behavior	No.	Cohen's *d*	*β*	95% CI Lower	95% CI Upper	T-value	P_{FDR}
**Frailty**	38,927	0.084	0.042	0.033	0.051	9.50	7.71×10^{-21}
**Overall health rating**	39,139	0.116	0.058	0.049	0.067	13.08	6.09×10^{-38}
**PHQ-4**	37,354	0.048	0.024	0.015	0.033	5.45	1.16×10^{-07}
**Health satisfaction**	39,112	0.092	0.046	0.037	0.055	10.50	5.61×10^{-25}
**PHQ-9**	26,617	0.039	0.019	0.009	0.030	3.66	3.43×10^{-04}
**Happiness with own health**	26,940	0.086	0.043	0.033	0.053	8.10	1.09×10^{-15}
**Neuroticism**	32,076	0.034	0.017	0.008	0.027	3.58	3.77×10^{-04}
**Easy to get up**	39,172	-0.020	-0.010	-0.018	-0.001	-2.22	2.70×10^{-02}
**PTSD symptom**	11,544	0.060	0.030	0.014	0.045	3.76	2.54×10^{-04}
**Falls in the last year**	39,150	0.058	0.030	0.021	0.038	6.80	1.04×10^{-11}
**Happiness**	39,070	0.032	0.015	0.007	0.024	3.64	3.77×10^{-04}

PHQ-4: patient health questionnaire-4; PTSD: post-traumatic stress disorder symptom.

Table S6. Results of the mediation analyses between frailty, health measures, and mean grey matter volume

	P_{FDR}	Prop. Mediated (%)	95% CI Lower-Upper		Total No.
Predictor: frailty; Response: behavioral outcomes; Mediator: grey matter volume					
PHQ-9	$<2\times10^{-4}$	0.263	0.107	0.477	27,322
PHQ-4	4.0×10^{-4}	0.369	0.193	0.586	38,375
Neuroticism	$<2\times10^{-4}$	0.454	0.228	0.74	32,937
Happiness	$<2\times10^{-4}$	0.502	0.262	0.813	40,090
Happiness with own health	$<2\times10^{-4}$	0.772	0.455	1.138	27,664
Health satisfaction	$<2\times10^{-4}$	0.885	0.599	1.202	40,130
Overall health rating	$<2\times10^{-4}$	0.941	0.673	1.245	40,163
PTSD symptom	$<2\times10^{-4}$	1.153	0.57	1.935	11,866
Easy to get up	$<2\times10^{-4}$	1.21	0.786	1.686	40,184
Falls in last year	$<2\times10^{-4}$	1.498	0.996	2.147	40,179
Predictor: behavioral outcomes; Response: frailty; Mediator: grey matter volume					
PHQ-9	5.2×10^{-3}	0.228	0.068	0.436	27,322
Neuroticism	4.4×10^{-4}	0.41	0.187	0.698	32,937
PHQ-4	$<2\times10^{-4}$	0.435	0.258	0.658	38,375
Happiness	$<2\times10^{-4}$	0.499	0.265	0.802	40,090
Happiness with own health	$<2\times10^{-4}$	0.885	0.561	1.262	27,664
Health satisfaction	$<2\times10^{-4}$	0.983	0.711	1.318	40,130
Overall health rating	$<2\times10^{-4}$	0.995	0.724	1.3	40,163
PTSD symptom	$<2\times10^{-4}$	1.185	0.599	1.984	11,866
Easy to get up	$<2\times10^{-4}$	1.402	0.978	1.911	40,184
Falls in last year	$<2\times10^{-4}$	1.586	1.083	2.174	40,179

We first investigated the association between physical frailty and 325 health-related measures and extracted the top-ten highly-correlated measures. Then, we examined the association of regional GMVs/tWMH with physical frailty and the top ten health measures. After that, for each of the ten health measures, we extracted the overlapped brain GMV that were significantly correlated with both frailty and the health measure of interest. For each health measure, we performed mediation analysis in R, where frailty was used as the independent variable, health measure as the dependent variable, and tWMH/mean GMV of brain regions that were significantly correlated with both frailty and the health measure constituted the mediator.

Table S7. Results of the mediation analyses between frailty, health measures, and white matter hyperintensities

	P_{FDR}	Prop. Mediated (%)	95% CI Lower-Upper		Total No.
Predictor: frailty; Response: behavioral outcomes; Mediator: white matter hyperintensities					
Easy to get up	0.30	0.189	-0.148	0.554	38,903
PHQ-9	2.2×10^{-2}	0.265	0.043	0.513	26,441
Neuroticism	2.2×10^{-2}	0.342	0.064	0.673	31,906
PHQ-4	8.0×10^{-4}	0.345	0.148	0.586	37,145
Happiness	2.2×10^{-2}	0.414	0.061	0.805	38,813
PTSD symptom	2.7×10^{-3}	0.622	0.202	1.275	11,465
Happiness with own health	$<2\times10^{-4}$	0.882	0.56	1.271	26,775
Health satisfaction	$<2\times10^{-4}$	1.04	0.746	1.391	38,851
Overall health rating	$<2\times10^{-4}$	1.182	0.869	1.535	38,883
Falls in last year	$<2\times10^{-4}$	1.299	0.808	1.87	38,898
Predictor: behavioral outcomes; Response: frailty; Mediator: white matter hyperintensities					
PHQ-9	5.0×10^{-4}	0.378	0.169	0.648	26,441
Easy to get up	1.4×10^{-2}	0.395	0.064	0.769	38,903
PHQ-4	$<2\times10^{-4}$	0.48	0.291	0.722	37,145
Neuroticism	5.0×10^{-4}	0.483	0.216	0.822	31,906
Happiness	5.0×10^{-4}	0.599	0.26	0.995	38,813
PTSD symptom	1.3×10^{-3}	0.631	0.21	1.279	11,465
Happiness with own health	$<2\times10^{-4}$	0.825	0.503	1.232	26,775
Health satisfaction	$<2\times10^{-4}$	0.985	0.694	1.329	38,851
Overall health rating	$<2\times10^{-4}$	0.986	0.681	1.313	38,883
Falls in last year	$<2\times10^{-4}$	1.396	0.922	1.976	38,898

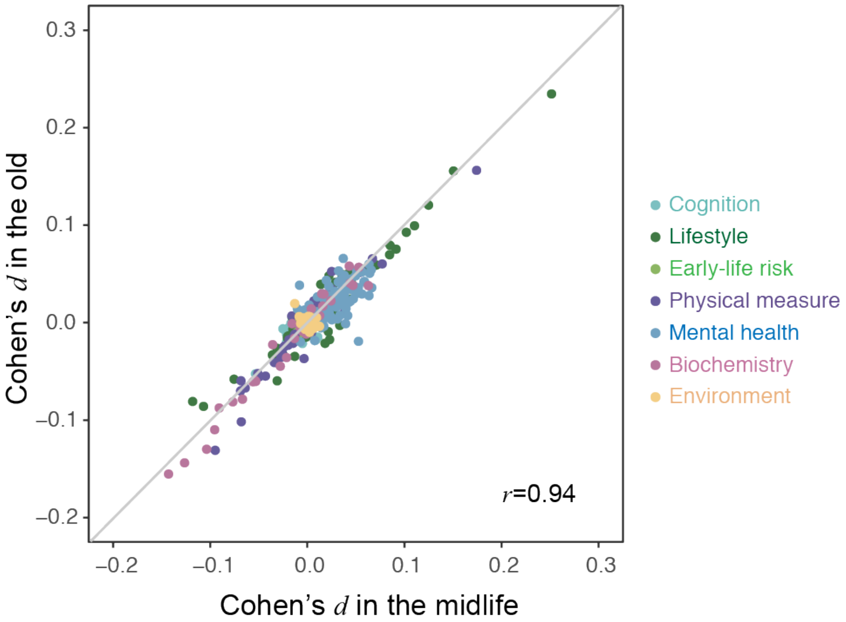

Figure S16. Associations between weight loss and health-related outcomes stratified by age. We reran association analysis to examine how weight loss relates to 325 health-related outcomes for middle-aged (45~60 years old) and older adults (>60 years old), separately. Results showed that the association patterns were highly similar between middle-aged and older adults (*r*=0.94).

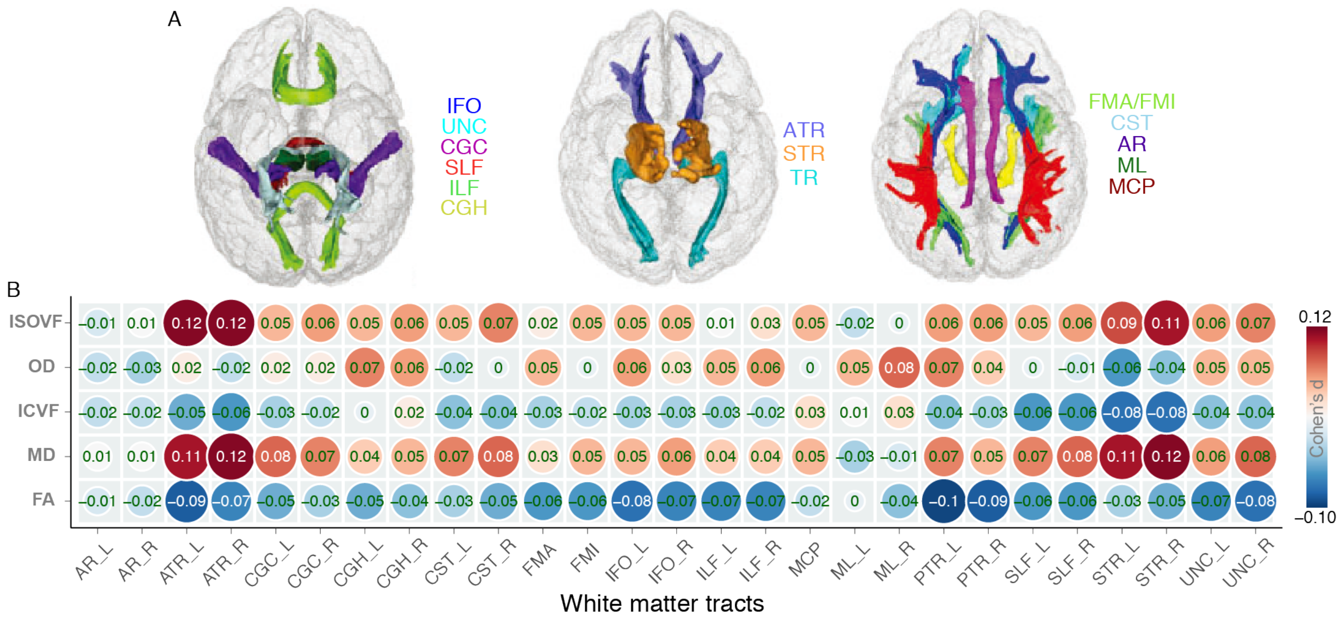

Figure S17. Associations between physical frailty and white matter microstructure indices across white matter tract regions while controlling for potential confounders. (**A**) White matter tracts of interest were generated using probabilistic tractography. This figure was adapted and modified from Cox et al.[13]. Imaging processing of DTI data can be found in [2].(**B**) Generally, the physical frailty showed primarily negative associations with regional FA and ICVF, but positive associations with MD, OD, and ISOVF. Specifically, white matter tracts showing the strongest associations mainly included FA in PTR, ATR, IFO, and UNC; MD in STR and ATR; ICVF in STR; OD in ML; ISOVF in ATR and STR.

Field IDs for these indices in UK Biobank were:

FA: 25488–25514;
MD: 25515–25541;
ICVF: 25650–25676;
OD: 25677–25703;
ISOVF: 25704–25730.

Abbreviation: FA: fractional anisotropy; ICVF: intracellular volume fraction; ISOVF: isotropic volume fraction; MD: mean diffusivity; OD: orientation dispersion; L: right; R: left; AR: acoustic radiation; ATR: anterior thalamic radiation; CGC, cingulate gyrus part of cingulum; CGH: parahippocampal part of cingulum; CST: corticospinal tract; FMA: forceps major; FMI: forceps minor; IFO: inferior fronto-occipital fasciculus; ILF: inferior longitudinal fasciculus; MCP: middle cerebellar peduncle; ML: medial lemniscus; PTR: posterior thalamic radiation; SLF: superior longitudinal fasciculus; STR: superior thalamic radiation; UNC: uncinate fasciculus.

Table S8. Baseline characteristic of participants in those with/without neuroimaging data at a 9-year follow-up

	Without neuroimaging data	With neuroimaging data	P for difference
Total N	441,174	41,859	
Age (mean ± sd)<br>range	56.65±8.12<br>38~73	55.01±7.54<br>40~70	<0.001
Sex, N/%			<0.001
Female	240,716/54.56	22,068/51.41	
Male	200,458/45.44	19,791/47.28	
Ethnicity, N/%			<0.001
White	416,822/94.48	40,535/96.84	
Non-White	24,352/5.52	1,324/3.16	
Body mass index (kg/m^2, mean ± sd)	27.48±4.81	26.55±4.22	<0.001
Waist-to-hip ratio (mean ± sd)	0.87±0.090	0.86±0.087	<0.001
Data acquisition date (range)	12/19/2006<br>10/01/2010	04/16/2007<br>10/01/2010	NA
Townsend deprivation index (mean ± sd)	-1.29±3.09	-1.88±2.73	<0.001
Average total household income, N/%			<0.001
Low (<£51,999)	284,153/64.41	24,484/58.49	
Middle (£52,000–£100,000)	74,561/16.90	10,874/25.98	
High (>£100,000)	19,737/4.47	3,020/7.21	
Unknown	62,723/13.71	3,481/8.32	
Education levels, N/%			<0.001
College/University	139,333/31.58	19,653/46.95	
Less than College	301,841/68.42	22,206/53.05	
Smoking status, N/%			<0.001
Never	238,457/54.05	25,414/60.71	
Ever	153,546/34.80	13,785/32.93	
Current	47,670/10.81	2,589/6.19	
Unknown	1,501/0.34	71/0.17	
Alcohol intake frequency, N/%			<0.001
Daily or almost daily	89,676/20.33	9,505/22.71	
Three or four times a week	100,721/22.83	11,757/28.09	
Once or twice a week	114,244/25.90	10,739/25.66	
One to three times a month	49,367/11.19	4,551/10.87	
Special occasions only	51,395/11.65	3,378/8.07	
Never	35,456/8.04	1,923/4.59	
Unknown	315/0.07	6/0.01	
Frailty severity, N/%			<0.001
0	239,773/54.35	27,318/65.26	
1	136,930/31.04	11,606/27.73	
2	44,982/10.20	2,388/5.70	
3	14,468/3.28	450/1.08	
4	4,427/1.00	86/0.21	
5	594/0.13	11/0.03	

The current study included 483,033 participants at the baseline visit, among whom 41,859 participants have MRI data collected at the imaging visit. Only participants with complete data available for frailty and basic demographic characteristics were analyzed here. Further, the samples with neuroimaging data were derived before performing any outlying data exclusion. Participants included in MRI scanning were more likely to be frail, have healthier behavior (but less likely to never or only occasionally drink alcohol), and have a higher socioeconomic status, indicating selection bias[14].

References

1. Petermann-Rocha F, Hanlon P, Gray SR, et al. Comparison of two different frailty measurements and risk of hospitalisation or death from COVID-19: findings from UK Biobank. *BMC medicine* 2020; **18**(1): 355.
2. Alfaro-Almagro F, Jenkinson M, Bangerter NK, et al. Image processing and Quality Control for the first 10,000 brain imaging datasets from UK Biobank. *Neuroimage* 2018; **166**: 400-24.
3. Gray JC, Thompson M, Bachman C, Owens MM, Murphy M, Palmer R. Associations of cigarette smoking with gray and white matter in the UK Biobank. *Neuropsychopharmacology* 2020; **45**(7): 1215-22.
4. Daviet R, Aydogan G, Jagannathan K, et al. Associations between alcohol consumption and gray and white matter volumes in the UK Biobank. *Nat Commun* 2022; **13**(1): 1-11.
5. Dekkers IA, Jansen PR, Lamb HJ. Obesity, brain volume, and white matter microstructure at MRI: a cross-sectional UK Biobank study. *Radiology* 2019; **291**(3): 763-71.
6. Antal B, McMahon LP, Sultan SF, et al. Type 2 diabetes mellitus accelerates brain aging and cognitive decline: Complementary findings from UK Biobank and meta-analyses. *eLife* 2022; **11**.
7. Jiang R, Westwater ML, Noble S, et al. Associations between grip strength, brain structure, and mental health in> 40,000 participants from the UK Biobank. *BMC medicine* 2022; **20**(1): 1-14.
8. Petermann-Rocha F, Lyall DM, Gray SR, et al. Associations between physical frailty and dementia incidence: a prospective study from UK Biobank. *The Lancet Healthy Longevity* 2020; **1**(2): e58-e68.
9. Hanlon P, Nicholl BI, Jani BD, Lee D, McQueenie R, Mair FS. Frailty and pre-frailty in middle-aged and older adults and its association with multimorbidity and mortality: a prospective analysis of 493 737 UK Biobank participants. *Lancet Public Health* 2018; **3**(7): e323-e32.
10. Hoogendijk EO, Afilalo J, Ensrud KE, Kowal P, Onder G, Fried LP. Frailty: implications for clinical practice and public health. *Lancet* 2019; **394**(10206): 1365-75.
11. Dent E, Martin FC, Bergman H, Woo J, Romero-Ortuno R, Walston JD. Management of frailty: opportunities, challenges, and future directions. *Lancet* 2019; **394**(10206): 1376-86.
12. Fried LP, Tangen CM, Walston J, et al. Frailty in older adults: evidence for a phenotype. *The journals of gerontology Series A, Biological sciences and medical sciences* 2001; **56**(3): M146-56.
13. Cox SR, Ritchie SJ, Tucker-Drob EM, et al. Ageing and brain white matter structure in 3,513 UK Biobank participants. *Nat Commun* 2016; **7**: 13629.
14. Batty GD, Gale CR, Kivimaki M, Deary IJ, Bell S. Comparison of risk factor associations in UK Biobank against representative, general population based studies with conventional response rates: prospective cohort study and individual participant meta-analysis. *BMJ* 2020; **368**: m131.
